# Fecal Microbiota Transplant from Human to Mice Gives Insights into the Role of the Gut Microbiota in Non-Alcoholic Fatty Liver Disease (NAFLD)

**DOI:** 10.3390/microorganisms9010199

**Published:** 2021-01-19

**Authors:** Sebastian D. Burz, Magali Monnoye, Catherine Philippe, William Farin, Vlad Ratziu, Francesco Strozzi, Jean-Michel Paillarse, Laurent Chêne, Hervé M. Blottière, Philippe Gérard

**Affiliations:** 1 Micalis Institute, Université Paris-Saclay, INRAE, AgroParisTech, 78350 Jouy-en-Josas, France; burzsd@gmail.com (S.D.B.); magali.monnoye@inrae.fr (M.M.); catherine.philippe@inrae.fr (C.P.); herve.blottiere@inrae.fr (H.M.B.); 2 Université Paris-Saclay, INRAE, MetaGenoPolis, 78350 Jouy-en-Josas, France; 3 Enterome, 75011 Paris, France; wfarin@enterome.com (W.F.); fstrozzi@enterome.com (F.S.); jmpaillarse@enterome.com (J.-M.P.); Lchene@enterome.com (L.C.); 4 INSERM UMRS 1138, Centre de Recherche des Cordeliers, Hôpital Pitié-Salpêtrière, Sorbonne-Université, 75006 Paris, France; vlad.ratziu@inserm.fr

**Keywords:** human, gut microbiome, high-fructose, high-fat diet, health, NAFLD, NAFL, liver

## Abstract

Non-alcoholic fatty liver diseases (NAFLD) are associated with changes in the composition and metabolic activities of the gut microbiota. However, the causal role played by the gut microbiota in individual susceptibility to NAFLD and particularly at its early stage is still unclear. In this context, we transplanted the microbiota from a patient with fatty liver (NAFL) and from a healthy individual to two groups of mice. We first showed that the microbiota composition in recipient mice resembled the microbiota composition of their respective human donor. Following administration of a high-fructose, high-fat diet, mice that received the human NAFL microbiota (NAFLR) gained more weight and had a higher liver triglycerides level and higher plasma LDL cholesterol than mice that received the human healthy microbiota (HR). Metabolomic analyses revealed that it was associated with lower and higher plasma levels of glycine and 3-Indolepropionic acid in NAFLR mice, respectively. Moreover, several bacterial genera and OTUs were identified as differently represented in the NAFLR and HR microbiota and therefore potentially responsible for the different phenotypes observed. Altogether, our results confirm that the gut bacteria play a role in obesity and steatosis development and that targeting the gut microbiota may be a preventive or therapeutic strategy in NAFLD management.

## 1. Introduction

Non-alcoholic fatty liver diseases (NAFLD) include different stages of liver damage, ranging from steatosis or non-alcoholic fatty liver, which are early phases (NAFL), to non-alcoholic steatohepatitis (NASH), which corresponds to an inflamed and sometimes fibrotic fatty liver. These diseases are among the main causes of hepatic cirrhosis and liver cancer (HCC) cases worldwide, mainly because of unhealthy diets and a sedentary lifestyle, all very prevalent in modern societies. NAFLD, which is associated with obesity and type II diabetes (T2D), is currently highly prevalent (up to a quarter to a third of Western and Asians populations) and therefore represents a major public health problem [1]. No pharmacological treatments are approved to stop or limit the progression of NAFLD, and management mainly relies on enforcing healthy dietary and lifestyle measures. Moreover, the contributors to and the dynamics of early disease phases are incompletely understood, and few preventive treatments are currently assessed.

The human intestinal microbiota consists of a set of microorganisms composed essentially of bacteria [2], archaea [3], protozoa, yeasts or mycobiota [4] and viruses or virobiota [5] that inhabit the digestive tract. Distributed in a differential way along the digestive tract, the microbiota presents a transverse spatial variability, in terms of number of species (richness) and their relative abundance (density), due to a specificity of adaptation to the different regions of the intestine, which each have specific physicochemical characteristics. Intestinal dysbiosis, i.e., imbalance of the microbiota, characterized by an increased abundance of pathogenic bacteria, a reduced diversity and losses of bacteria considered beneficial, has been described in many pathologies involving different human organs. Regarding liver diseases, a dysbiosis has been identified in patients at different stages of NAFLD, although discrepancies between the different studies do not allow defining a clear NAFLD gut microbiota profile. Decreased abundance of Bacteroidetes and *Ruminococcaceae* and increased abundance of *Lactobacillaceae*, *Veillonellaceae* and *Dorea* are the most frequently reported [6]. Moreover, some gut bacteria have been associated with different stages of the liver disease, with increased levels of *Bacteroides* correlating with NASH, while increased *Ruminococcus* abundance was found correlated with fibrosis development [7]. Using whole-genome shotgun sequencing, Loomba et al. further characterized the gut microbiome composition of NAFLD patients which provided preliminary evidence for a fecal microbiome-derived metagenomic signature allowing to detect advanced fibrosis in NAFLD [8]. Finally, we recently showed that nonvirulent endotoxin-producing strains of pathogenic species (*Enterobacter cloacae*, *Escherichia coli* and *Klebsiella pneumoniae*) overgrowing in the obese human gut can act as causative agents for induction of NAFLD [9]. The mechanisms by which the gut microbiota may contribute to NAFLD include dysbiosis-induced gut permeability, endotoxemia, endogenous production of ethanol, increased energy harvest from food and choline and bile acid metabolisms [10]. Recently, the bacterial metabolism of branched chain and aromatic amino acids has also been implicated and derived microbial metabolites including 3-(4-hydroxyphenyl)lactate and phenylacetic acid have been associated with steatosis and fibrosis development [11,12].

Fecal microbiota transplant (FMT) can be used to demonstrate causality or to treat patients suffering from a disease involving the gut microbiota. Recently, FMT from a carefully selected healthy donor was reported to reduce hospitalizations and to improve cognition and dysbiosis in patients suffering from cirrhosis with recurrent hepatic encephalopathy [13]. Moreover, using manipulation of the gut microbiota, animal studies began to demonstrate its direct roles in the appearance and development of the diverse liver lesions observed in NAFLD. We were the first to demonstrate that the gut microbiota has a major contributing role in the development of steatosis induced by a high-fat diet using gut microbiota transplant from mouse to mouse [14], and similar studies further revealed the contribution of the microbiota in NASH [15] and fibrosis [16]. However, causality data involving the microbiota in the disease remain scarce in the literature. Transplantation of the human microbiota to germ-free or antibiotic-treated mice has been used for decades and is now considered the best model to both prove causality and elucidate mechanisms linking the microbiota to disease development that cannot be achieved in humans [17]. In the context of liver diseases, this approach has been successfully used by us and others to decipher the involvement of the gut microbiota in both NAFLD and alcoholic liver disease [9,12,18].

In the present study, our aim was to determine whether the transfer of the gut microbiota from a patient affected by NAFL could favor the induction of the disease in specific mice models. Here, we limited our investigations to NAFL, the early stages of the disease, characterized by abnormal fat excess in the liver, in order to understand how the intestinal microbiota can influence the early onset of the disease. The underlying hypothesis was that the transfer of the microbiota from an NAFL patient would worsen the fatty liver disease in recipient mice as compared with the transfer of the microbiota from a healthy individual. This study sheds additional light on the impact of the gut microbiota on the establishment of the natural pathophysiology of NAFLD, especially during the early phases of the disease.

## 2. Materials and Methods

### 2.1. Clinical Cohort

A cohort of 20 NAFLD patients with moderate obesity, average age of 59, was recruited; a liver biopsy determined a diagnosis of NAFL or NASH. All subjects gave their informed consent for inclusion before they participated in the study. The study was conducted in accordance with the Declaration of Helsinki, and the protocol 2016-A01074-47 was approved in January 2017 by the French Ethics Committee of CPP (*Comité de Protection des Personnes*)-Ile de France VI. Only one patient was diagnosed with NAFL and was thus selected. A healthy individual of the same sex, of neighboring age, with similar plasma AST (aspartate transaminase), ALT (alanine transaminase) and triglycerides was paired with this NAFL patient. The NAFL patient differs from the healthy individual by a tendency to insulin resistance (homeostasis model assessment of insulin resistance (HOMA-IR) close to 2), overweight and steatosis reaching 60% (Table 1). Within one month after the biopsy, the stools of the individuals were collected, aliquoted and preserved in a conservation solution as previously described [19].

### 2.2. Animal Experimentation

**Figure 1:**
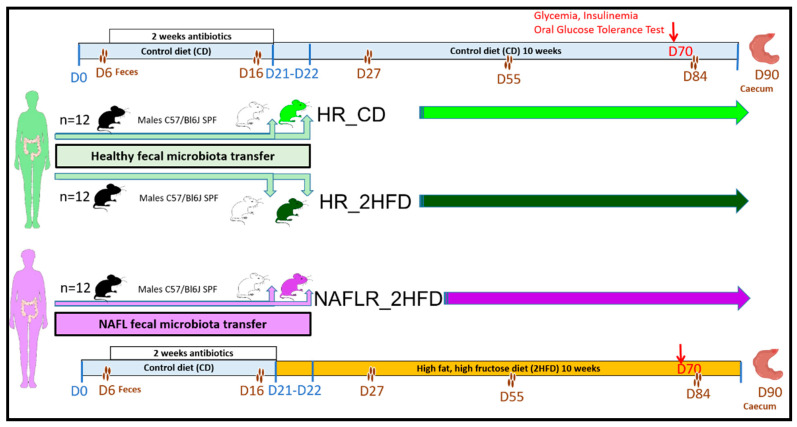
Design of the mouse experiment. The experiment was performed on three groups each composed of 12 specific pathogen-free (SPF) C57BL/6J male antibiotic-treated mice: (1) healthy microbiota receiver on control diet (HR_CD) group; (2) healthy microbiota receiver on high-fructose, high-fat diet (HR_2HFD) group; (3) NAFL microbiota receiver on 2HFD (NAFLR_2HFD) group. HR, healthy human microbiota receiver mice; CD, control diet; 2HFD, high-fructose, high-fat diet; NAFLR, NAFL patient microbiota receiver mice; green woman, healthy microbiota donor; purple woman, NAFL microbiota donor; black mice, SPF mice; white mice, antibiotic-treated mice; light green mice, HR_CD mice, HR mice on CD; dark green mice, HR_2HFD mice, HR mice on 2HFD; purple mice, NAFLR_2HFD mice, NAFLR mice on 2HFD. Four brownish dots, mice feces harvest; D, day; D0, mice arrival; D6, basal SPF mice feces harvest; D16, feces harvest after 2 weeks antibiotic treatment; D21-D22, two human fecal microbiota transplants (FMTs) at 24-h intervals; D27, one week after FMT; D55, one month after FMT; D70, 7 weeks of 2HFD treatment, glycemia, insulinemia and oral glucose tolerance test; D84, two months after FMT; D90, 10 weeks of 2HFD treatment; SPF, specific pathogen-free.

Procedures were performed according to the European Guidelines for the Care and Use of Laboratory Animals and approved by the French Veterinary Authorities (Authorization number 78–60). The experimental protocol (Figure 1) was agreed upon by the French *Ministère de l’Education Nationale, de l’Enseignement Supérieur et de la Recherche* (APAFIS#18425-2018110521086756 v2).

Thirty-six specific pathogen-free (SPF) C57BL/6J male mice, 7 weeks old, were purchased from Charles River Laboratories, France, from 36 different litters. C57BL/6J mice were chosen due to their well-described susceptibility to diet-induced metabolic disorders. From weaning, they were maintained in 9 different cages (colors in Supplementary Figure S1b). On arrival, animals were housed under controlled conditions of temperature, hygrometry and 12-h light/dark cycle in an SPF facility at INRAE, Jouy-en-Josas (Unité Expérimentale d’Infectiologie des Rongeurs et Poissons). They were individually weighted, microchipped and randomly housed in 9 new cages, 4 mice per cage; they received a conventional γ-irradiated 45 kGy mice control diet (CD), SAFEA03 R03-40, *ad libitum* (3.24 kcal/g: 14% energy from fat, 25% energy from proteins, 61% energy from carbohydrates) (SAFE, Augy, France, CD, Table S1). On day 6 after arrival, feces of the mice were individually harvested (basal microbiota). Then, they received in the autoclaved drinking water for 2 weeks a mixture of broad-spectrum antibiotics (Vancomycin 45 µL/mL, Streptomycin 1 mg/mL, Colistin 1 mg/mL and Ampicillin 1 mg/mL) *ad libitum*, to deplete their endogenous microbiota [20]. The irradiated litter and food were replaced every 48 h, and each cage with a filter cover was autoclaved once a week. On day 16, an all-bacteria qPCR was performed to verify complete absence of detectable bacteria in the feces (threshold of 99.99% depletion was considered to be similar to germ-free mice). On day 20, the mice were randomly assigned to three experimental groups of 12 mice, 4 microchipped mice per cage, using an in-house R software script to randomize cages depending on mice body weight (Figure S1a) and on occupancy by the provider (Figure S1b). Three iterations of randomization were needed to balance the dataset (Figure S1a,b). The stools of the selected human individuals (NAFL patient and healthy individual) were inoculated (200 µL per mouse) by two gavages or FMT at 24-h intervals (day 21 and 22, Figure 1). Fecal transplants contained more than 70% of viable bacteria as shown by flow cytometry (Figure S2). The first group of mice received fecal microbiota from the healthy individual and was maintained for 10 weeks on CD and was referred to as “healthy microbiota receiver on CD, HR_CD” (Figure 1). The second group of mice also received the fecal microbiota from the healthy individual. The third group of mice received the fecal microbiota from the NAFL patient (Figure 1). From day 21 to the end of the experiment, these last two groups were fed *ad libitum* for 10 weeks an experimental high-fat, high-fructose diet (2HFD), to induce NAFLD, consisting of a γ-irradiated 45 kGy high-fat diet, D12492 (HFD, Table S1), containing 31.7% lard and 3.2% soybean oil (5.24 kcal/g: 60% energy from fat, 20% energy from protein, 20% energy from carbohydrate, Research Diets, Lynge, Denmark) associated with a 30% (*w*/*v*) final high-fructose (HF) drinking solution (1.2 kcal/mL). The HF solution was made by dissolution of D(-)-Fructose > 99.5% purchased from Roth Sochiel (Lauterbourg, France) in autoclaved tap water, and the final solution was then sterilized through 0.22 µm filtration. The experimental combination of HFD + HF was called the high-fructose, high-fat diet (2HFD). The second group of mice was referred to as “healthy microbiota receiver on 2HFD, HR_2HFD”. The third group of mice was subsequently referred to as “NAFL microbiota receiver on 2HFD, NAFLR_2HFD”. Body weight and food and liquid consumption were monitored weekly.

Mice feces were collected individually at five time points: on days 6 (basal), 16 (after two weeks of antibiotic treatment), 27 (one week after FMT), 55 (one month after FMT) and 84 (two months after FMT). Mice were euthanized on day 90, 10 weeks after FMT and 2HFD treatment. Liver, epididymal and mesenteric adipose tissues, the caecum and blood were then harvested (Figure 1).

### 2.3. Preparation and Preservation of Fecal Transplants

A preservation solution in maltodextrin-trehalose diluent (MD) in the ratio of 3:1 was furnished by MaaT Pharma through material transfer agreement (Lyon, France). Fecal transplant was prepared as previously described [19]. Briefly, for each donation, the Bristol stool scale was evaluated, and the stool was weighed. On one hand, 10 g of feces was transferred into Stomacher^®^ Filter Bags (Seward BA6041/8TR, or VWR 432-3119, 0.5 mm holes) within the anaerobic chamber. Four mL of diluent was added per g of stool. Five-min hand mixing through the filter bags (closed by clips, VWR 432-3116) ensured both homogenization and filtration. Then, the suspension was aliquoted and stored in a −80 °C freezer. Thawing was carried out for 5 min in a water bath at 37 °C. On the other hand, to ensure good fecal samples collection and conservation, we followed the International Human Microbiome Standards (IHMS; http://www.microbiome.standard.org/) consortium IHMS SOP05 [21], an international consensus on human fecal samples self-collection, giving a step-by-step description in the case that the samples will be handled within 7 days and preserved in a stabilizing solution during transportation.

### 2.4. 16S rRNA Sequencing Analysis

DNA was extracted from feces or caecum contents using the Gnome DNA Isolation Kit (MP Biomedicals, Santa Ana, CA, USA). Quantifications of total bacteria DNA were performed by real-time qPCR following the procedure previously described by Furet et al. [22]. A total of 220 DNA samples, corresponding to the two inocula from the healthy human donor, two inocula from the NAFL human donor used for inoculation, 180 fecal samples at five sampling timepoints and 36 caecal samples from recipient mice, were analyzed. The V3-V4 region of the 16S rRNA genes was amplified using MolTaq (Molzym, Bremen, Germany) and the following primers: V3PCR1F_460bp: 5′-CTTTCCCTACACGACGCTCTTCCGATCTACGGRAGGCAGCAG-3′; and V4PCR1R_460bp: 5′-GGAGTTCAGACGTGTGCTCTTCCGATCTTACCAGGGTATCTAATCCT-3′ [23]. Purified amplicons were sequenced using the MiSeq sequencing technology (Illumina, San Diego, CA, USA) on the GeT-PLaGe platform (Genotoul, Toulouse, France). Paired-end reads obtained from MiSeq sequencing were analyzed using the Galaxy-supported pipeline named Find, Rapidly, Operational Taxonomic Units (OTUs) with Galaxy Solution (FROGS) [24]. For the preprocessing, reads with length between 380 and 500 bp were retained. The clustering and chimera removal tools followed the guidelines of FROGS [24]. Assignment was performed using the Silva 132 database updated in December 2017 with the top-quality pintail 100 (https://www.arb-silva.de/). OTUs with abundances lower than 0.005% were removed from the analysis [25]. 16S sequencing data were analyzed using the Phyloseq, DESeq2 and ggplot2 R packages in addition to custom scripts [26]. Samples were rarefied to even sampling depths before computing within-samples compositional α-diversity (observed richness and according to the bias-corrected Chao1 estimator, or other indicators, Figure S4d) and between-samples compositional β-diversity (Unifrac, Bray-Curtis, Jaccard). Alpha diversity data were analyzed using unpaired Student’s t test in a 2 by 2 comparison. Principal coordinate analysis was performed as previously described [27]. A permutational multivariate ANOVA test was performed on the Unifrac matrices using 9999 random permutations and at a significance level of 0.01. Phylum relative abundances were compared using the Kruskal–Wallis test, followed by Dunn’s test because they did not satisfy the normal assumption of the standard ANOVA. Family-level abundances were compared 2 by 2 using the Mann–Whitney test. Raw, unrarefied OTU counts were used to produce relative abundance graphs and to find taxa with significantly different abundances in HR_2HFD and NAFLR_2HFD. A negative binomial model was fit to each OTU, using DESeq2 [28] with default parameters, to estimate abundance log-fold changes (FCs). Values of p were corrected for multiple testing using the BH procedure to control the false discovery rate and significant OTUs were selected based on effect size (FC > 8 or FC < 1/8), adjusted *p*-value (<0.001) and prevalence (relative abundance > 0.1% in at least half the samples of a group).

### 2.5. Shotgun Sequencing Analysis

Fecal DNA extraction was carried out according to the IHMS consortium SOP07 [29]. Shotgun metagenomic sequencing was carried out by the provider Eurofins (Ebersberg, Germany) (ISO certified) on the NovaSeq 6000 platform (Illumina). Reads were cleaned using Trimmomatic with a sliding window of 4 nucleotides, trimming a read when the Phred quality score dropped below 20. Cleaned reads were subsequently filtered from human-derived sequences by performing an alignment on the human genome (hg19) using BBmap with 95% identity. All reads positively aligned were discarded and not retained for downstream analysis. The gene abundance profiling was based on the MetaHit catalogue of reference genes in the human gut microbiome (3.3 M genes [30]). Filtered high-quality reads were mapped to the 3.3 M gene catalogue using BBmap, retaining only uniquely mapped reads of at least 45 nucleotides and with a minimum of 95% identity. Gene counts were derived from the number of mapped reads and then normalized according to the gene length and the total number of mapped reads per sample. Data were then additionally aggregated at different taxonomical levels (i.e., species, genus, family, phylum) according to the 3.3 M catalogue annotation information. All statistical analyses were performed using R software.

### 2.6. Short-Chain Fatty Acids Quantification in Caecal Contents

Measurement of short-chain fatty acids (SCFA) and branched chain fatty acids (BCFA) from mice caecal contents was performed using gas chromatography as previously described by Lan et al. [31], with slight modifications regarding the gas chromatograph equipment and the carrier gas. The analyses were performed on an Agilent (Les Ulis, France) gas chromatograph (7890) equipped with a split/splitless injector 7650 and ionization flame detector. Carrier gas (H_2_) flow rate was 10 mL/min and inlet, column and detector temperatures were 200, 100 and 240 °C, respectively. Data were collected and peaks were integrated using OpenLab Chem station software (Les Ulis, France).

### 2.7. Plasma Assays

On day 90, blood was collected from mice before euthanasia by submandibular puncture into tubes containing 5 µL EDTA 0.5 mol/L. After centrifugation (6000× *g*, 20 min, 4 °C), plasma was aliquoted and frozen at −80 °C until further analysis. Measurements of plasma AST, ALT, triglycerides (TG), high-density lipoprotein cholesterol (HDL), total cholesterol and ferritin were performed on the biochemistry platform (CRI, UMR 1149, Paris) with an Olympus AU400 Chemistry Analyzer. LDL (low-density lipoprotein) cholesterol was calculated according to the Friedewald formula:

LDL-Cholesterol = Total Cholesterol − HDL-Cholesterol − TG/2.2 (mmol/L), with all TG < 4.6 mmol/L.

Measurements of non-fasting plasma insulin and leptin were performed using a mouse-specific insulin and leptin ELISA Kit (Merck Millipore, Burlington, MA, USA).

### 2.8. Metabolomic Profile

A targeted metabolomic analysis was performed on the analytical chemistry platform (LABERCA Oniris, Nantes, France) using the MxP500 Quant kit (Biocrates Life Science AG, Innsbruck, Austria). It is a commercially available assay, which was originally developed for human plasma, covering 630 metabolites from 14 metabolite and 12 lipid classes. Metabolite detection relies on two MS methods. First, an LC-MS/MS method is used to detect alkaloids, amine oxides, amino acids, amino acid-related metabolites, bile acids, biogenic amines, carboxylic acids, cresols, fatty acids, hormones, indole derivatives, nucleobase-related metabolites, vitamins and cofactors, and then FIA-MS/MS is used for detection of acylcarnitines, ceramides, cholesterol esters, diacylglycerols, dihydroceramides, glycerophospholipids, glycosylceramides, sphingolipids, triacylglycerols and sugars. The kit provided either quantitative measurements using seven-point calibration levels including isotopically labeled internal standards for LC-MS/MS or one-point calibration for FIA-MS/MS. The rest of the metabolites were measured semi-quantitatively, i.e., standards with similar chemical properties to the targets were used. Sample preparation was performed according to the kit protocol. For LC-MS/MS analysis, 150 µL of the samples was transferred and diluted with 150 µL H_2_O on an empty plate, and for FIA-MS/MS analysis, 250 µL of the FIA mobile phase (made by mixing 290 mL MeOH and a 10 mL ampule Biocrates FIA mobile phase additive, provided with the kit) was added directly to the samples on the collection plate. The extracts were analyzed using an EXIONLC Ultra High Pressure Liquid Chromatography System coupled in-line to a 6500+ Q-Trap LC/MS system (Sciex, Framingham, MA, USA). The FIA-MS/MS method was carried out on the same MS system. The instrumental analysis was performed according to the guidelines from the manufacturer. Briefly, the analysis was conducted in the positive and negative ionization modes for both LC-MS/MS and FIA-MS/MS. The chromatography was conducted using the UHPLC column provided with the kit. Data were collected using Analyst software v1.7 (Sciex, Framingham, MA, USA) and analyzed with Biocrates MetIDQ software v8.7.1 DB110 Oxygen 2893 (Biocrates Life Sciences AG, Innsbruck, Austria). Internal standards and quality control samples of the MxP500 Quant Kit were used to benchmark the quality of the assay, the robustness of the data and the calculation for the concentrations of all the metabolites detected.

### 2.9. Liver Histology and Scoring

A slice of the liver’s left lobe was fixed in 4% paraformaldehyde for 24 h and then transferred into ethanol, fixed in paraffin, processed, sectioned into slices approximately 5 µm thick, mounted on a glass slide and stained with hematoxylin-eosin. Then, the slides were scanned (Pannoramic Scan 3DHistech slide scanner, Histology facilities @BRIDGe, GABI, INRAE, AgroParisTech, Paris-Saclay University, Paris, France). Slices were scored for steatosis and inflammation by two experienced pathologists mastering this evaluation and blinded to the experiment. Five fields per liver section were analyzed (objective magnification 20×) using two criteria: steatosis and inflammation according to Brunt’s scoring [32]. Hepatic steatosis was assessed using a score ranging from 0 to 3 depending on the amount of lipids accumulated inside the hepatocytes. A score of 0 was assigned to sections where lipids represented less than 5% of the cell and a score of 3 was assigned to those where lipid area occupied more than 66% of the cell surface. Inflammation of the tissue was assessed using a score ranging from 0 to 3. These scorings were attributed according to the number of inflammatory foci observed in a field. In absence of foci, the score was noted as 0; in the presence of more than four foci, the score was noted as 3 [32].

### 2.10. Hepatic Triglycerides (TG) Measurement

A portion (30–70 mg) amounting to approximately 1/4 of the left liver lobe was frozen in liquid nitrogen and stored at −80 °C until TG extraction. Samples were homogenized in chloroform–methanol (2/1; *v*/*v*) in order to extract total lipids according to the Folch method [33]. The organic extract was dried and dissolved in isopropanol. The TG content was measured using a TG determination kit (Sigma-Aldrich, Saint-Louis, MI, USA), according to the manufacturer’s instructions and expressed in mmol of TG per gram of liver.

### 2.11. Real-Time Quantitative Polymerase Chain Reaction (qPCR)

A 1/4 portion of the left liver lobe and the empty caecum were stored in RNAlater^TM^ stabilization solution (Invitrogen, Carlsbad, CA, USA) at −80 °C until further analysis. Total RNA was extracted from the liver with the RNAeasy Plus Mini Kit (Qiagen, Hilden, Germany). RNA integrity and concentration were checked with RNA 6000 Nano chips on an Agilent 2100 bioanalyser (Agilent Technologies, Amsterdam, The Netherlands). Total RNA (10 µg per reaction) was reverse-transcribed into complementary DNA using a high-capacity cDNA reverse transcription kit (Applied Biosystems, Thermofisher Scientific, Foster City, CA, USA) according to the manufacturer’s instructions. Real-time qPCR was performed on an Applied biosystem step one plus machine. The relative gene expressions were normalized to two housekeeping genes: gapdh and actb (glyceraldehyde-3-phosphate dehydrogenase, actin beta), chosen based on results obtained from TaqMan mouse endogenous control arrays (Applied biosystems). The HR_CD samples were used as reference for the relative expression of genes.

### 2.12. Oral Glucose Tolerance Test (OGTT)

Fasting glycemia and insulinemia measurements as well as OGTT were performed on day 70, i.e., after 7 weeks of the 2HFD regimen, 21 days before euthanasia (Figure 1). After 6 h of fasting, a glucose solution (2 g glucose/kg body weight) was administered by oral gavage. Blood glucose levels at time 0 (fasting glycemia, determined before glucose gavage) and 15, 30, 60, 90 and 120 min after glucose gavage were analyzed using an Accu-Check glucometer (Roche, Meylan, France). The glucose levels were plotted against time, and the AUC (area under curve) was calculated. The plasma insulin concentrations at time 0 (fasting insulinemia) and after 30 min were assayed in venous blood (collected in EDTA-coated tubes), harvested from the marginal tail vein, using a mouse-specific Insulin ELISA Kit (Merck Millipore, St Quentin-en-Yvelines, France). Insulin resistance was estimated by homeostasis model assessment of insulin resistance (HOMA-IR index) and calculated according to the following formulas:

HOMA-IR (fasting) = fasting glucose (mmol/L) × fasting insulin (mU/L)/22.5.

HOMA-IR (non-fasting) = non-fasting glucose (mmol/L) × non-fasting insulin (mU/L)/22.5.

### 2.13. Statistical Analysis

Dataset normality was tested using the D’Agostino and Pearson normality test. Normally distributed data with equal group variances were expressed as mean ± standard errors of the mean (SEM). Non-normally distributed data, or those belonging to unequal group variances, were expressed as medians (interquartile ranges). The level of significance was set at *p* < 0.05 (* *p* < 0.05, ** *p* < 0.01, *** *p* < 0.001). Statistical comparisons performed for diet impact (HR_CD vs. HR_2HFD) and for microbiota impact (HR_2HFD vs. NAFLR_2HFD) used the subsequent unpaired Student’s t test or unpaired Mann–Whitney test. Calculations were performed with R 3.5 software and GraphPad Prism software (version 7.00, La Jolla, CA, USA).

## 3. Results

### 3.1. Assessment of Human Microbiota Transfer to Mice

**Figure 2:**
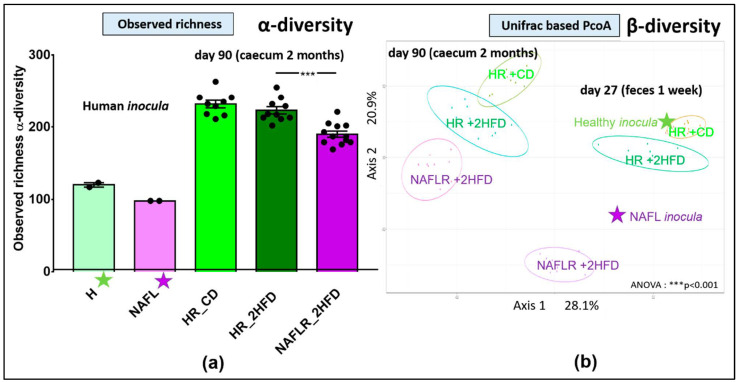
16S rRNA *inocula*, fecal and caecal microbiome analysis. (**a**) Observed richness, *n* = 12 mice/group, *** *p* < 0.001 for microbiota statistical impact (NAFLR_2HFD vs. HR_2HFD). (**b**) Unifrac-based PcoA, ANOVA: *** *p* < 0.001. PcoA, principal coordinate analysis; green star, healthy human microbiota; purple star, NAFL human microbiota; HR, healthy human microbiota receiver mice; CD, control diet; 2HFD, high-fructose, high-fat diet; NAFLR, NAFL patient microbiota receiver mice; HR_CD, HR mice on CD; HR_2HFD, HR mice on 2HFD; NAFLR_2HFD, NAFLR mice on 2HFD; day 27, one week after FMT; day 90, 10 weeks after FMT.

A patient biopsied with NAFL and a healthy (H) individual matched for age and sex were selected (see Materials and Methods 2.1. Clinical cohort for details). Inocula for mice gavage were prepared from their respective stool samples (Figure 1). The microbial taxonomic fingerprints (16S rRNA) were analyzed on human stool samples, and then the transfer efficiency in recipient mice (NAFLR, HR) was checked on feces one week after FMT (D27), one month after FMT (D55) and then on caecal contents 10 weeks after FMT (D90). The two inocula, used for the two consecutive gavages, from the healthy individual showed a higher α-diversity (observed richness: 120, Chao1: 169.9) and therefore a higher richness of OTUs (Figure 2a, Figure S3a) than the two inocula from the NAFL patient (observed richness: 98, Chao1: 102.7). The inocula from the NAFL patient showed a lower α-diversity (Figure 2a, Figure S3a) than the caecal samples from the recipient mice of NAFLR (observed richness: 190.1 ± 4.3, Chao1: 227.9 ± 8.9). Similarly, inocula from the healthy individual showed a lower α-diversity (Figure 2a) in comparison with the recipient mice of HR under the CD (observed richness: 231.8 ± 5.3, Chao1: 275.3 ± 6.2) or 2HFD (observed richness: 223.1 ± 5.1, Chao1: 268.1 ± 9.4). We computed the unweighted Unifrac principal coordinate analysis (PcoA) to look for dissimilarities between the two inocula and the fecal (day 27) and caecal (day 90) microbiomes of recipient mice. ANOVA showed significant phylogenetic separation of microbiota ellipses from recipient mice of HR_CD vs. HR_2HFD vs. NAFLR_2HFD, which remained close to their respective inocula one week after FMT (day 27) and evolved in parallel ways until the end of the experiment, in the caecum (day 90, Figure 2b).

The healthy human inocula (green star) and the NAFL human inocula (purple star) were split as revealed by β-diversity, indicating distinct microbiota compositions. The HR mice microbiome (green ellipses) clustered with the healthy inocula, whereas the NAFLR mice microbiome (purple ellipse) clustered with the NAFL inocula (Figure 2b). Other β-diversity evaluation methods (Bray–Curtis, Jaccard and weighted Unifrac) showed similar separations in terms of variance and dispersion of the groups (not shown). Differences between the NAFL and healthy inocula are characterized in the NAFL by an increase in the abundance of *Ruminococcaceae* (Firmicutes phylum) and *Tannerellaceae* (Bacteroidetes phylum) and a decrease in the abundance of *Desulfovibrionaceae* (Proteobacteria phylum) and *Rikenellaceae* (Bacteroidetes phylum). These differences were transferred in the NAFLR_2HFD and HR_2HFD mice, respectively (Figure S3b). At the genus level, these human to mice transferred differences are represented by *Ruminoclostridium 9* (*Ruminococcaceae* family) and *Parabacteroides* (*Tannerellaceae* family) being more abundant in NAFLR_2HFD than in HR_2HFD and *Alistipes* (*Rikenellaceae* family) being less abundant (Figure S3c). At the family level, the mouse basal microbiota on day 6 was in particular characterized by a high abundance of the *Muribaculaceae* family (formerly S24-7), known to be dominant in the mouse gut microbiota [34] and not present in the healthy or NAFL *inocula*. One week following inoculation with human FMT, no more *Muribaculaceae* were detected in mice feces, suggesting a good humanization of the mice microbiota (Figure S4).

We further validated our transfer one month after FMT (day 55) by shotgun sequencing of the mice feces and compared them with the human feces before preservation and the inocula. For all samples, more than 5 million paired-end reads were obtained and the mapping rate on the human 3.3 M gene catalogue [35] was above 75%, except for mice samples for which the mapping rate was between 40 and 60%. Preparation and preservation have an impact on some species of the human microbiome, as described previously [19], but inocula and human feces were similar in terms of relative abundance (not shown). Gene richness was about 300,000 for human feces and slightly lower, 278,000 genes, for inocula. However, mice fecal gene richness was low (128,000 genes) due to the lower mapping on the human gene catalogue. Thus, shotgun sequencing confirmed that one month after FMT, the mice microbiota differed from that of human feces and inocula. This suggests that FMT from human to mice, followed by one-month 2HFD treatment, had a huge impact on the bacterial ecology found in mice in comparison with the inocula ecology. At the phylum level, this led to an increase in Verrucomicrobia in mice feces, while abundances of Bacteroidetes and Firmicutes were found similar in human samples and recipient mice.

### 3.2. Impact of the 2HFD Regimen (HR_CD vs. HR_2HFD)

In order to assess the impact of the experimental diet alone, a diet rich in fructose and fat for 10 weeks (2HFD), we compared mice inoculated with the healthy microbiota, i.e., from the healthy donor, that were fed a control diet (CD) or the experimental diet (2HFD).

#### 3.2.1. Body Weight Gain

**Figure 3:**
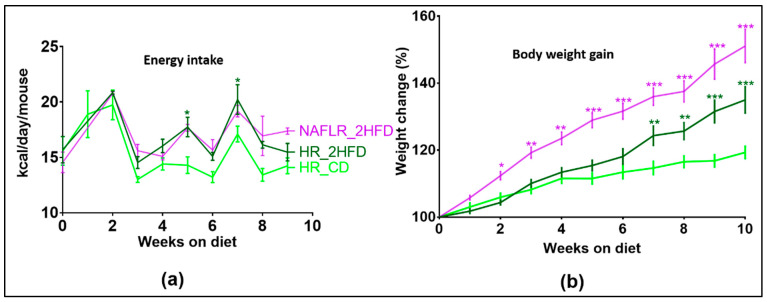
(**a**) Food energy intake (kcal/day/mouse), *n* = 12 mice/group and (**b**) mice body weight gain (%) follow-up, *n* = 12 mice/group. HR, healthy human microbiota receiver mice; CD, control diet; 2HFD, high-fructose, high-fat diet; NAFLR, NAFL patient microbiota receiver mice; HR_CD, HR mice on CD; HR_2HFD, HR mice on 2HFD; NAFLR_2HFD, NAFLR mice on 2HFD; green (* *p* < 0.05, ** *p* < 0.01, *** *p* < 0.001) is used for diet impact (HR + 2HFD vs. HR + CD) statistical comparisons and purple (* *p* < 0.05, ** *p* < 0.01, *** *p* < 0.001) is used for microbiota impact (NAFLR_2HFD vs. HR_2HFD) statistical comparisons.

Mice fed with the experimental diet HR_2HFD had a higher energy intake (Figure 3a) and gained more weight after seven weeks on this diet (with a mean increase difference in body weight of 9.7% at week 7, *p* = 0.003, and of 15.7% at week 10, *p* < 0.001, Figure 3b) compared to HR_CD controls. At end of the experiment, HR_2HFD mice had higher body weight than HR_CD mice (35.0 g ± 0.8 vs. 29.5 g ± 1.2, respectively, *p* < 0.001). These body weight differences were explained by more epididymal (2.2 g ± 0.3 vs. 0.9 g ± 0.04, respectively, *p* < 0.001, Figure S5a) and mesenteric adipose tissues (1.9 g ± 0.13 vs. 1.4 g ± 0.07, respectively, *p* < 0.01, Figure S5b), while liver weights were slightly lower in HR_2HFD mice than in HR_CD mice (4.2 g ± 0.1 vs. 4.8 g ± 0.1, respectively, *p* < 0.05, Figure S5c). Compared to the CD, the HR caecum index, defined as caecum weight/mice final body weight, was lower under the 2HFD regimen (0.77 ± 0.046 vs. 2.2 ± 0.16%, respectively, *p* < 0.001, Figure S6a).

#### 3.2.2. Plasma Assays

**Figure 4:**
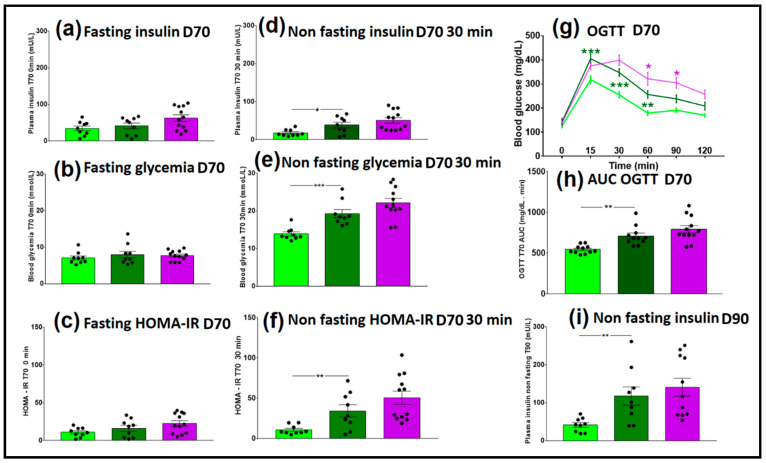
Glycemic and insulin resistance parameters of CD- and 2HFD-fed HR vs. NAFLR mice, *n* = 9–12 mice/group. (**a**) Fasting insulinemia at D70, t = 0 min Oral glucose tolerance test (OGTT); (**b**) fasting glycemia at D70, t = 0 min OGTT; (**c**) fasting Homeostasis Model Accessment of insuline resistance (HOMA-IR) at D70, t = 0 min OGTT; (**d**) non-fasting insulinemia at D70, t = 30 min OGTT; (**e**) non-fasting glycemia at D70, t = 30 min OGTT; (**f**) non-fasting HOMA-IR at D70, t = 30 min OGTT; (**g**) blood glucose follow-up during OGTT at D70; green (** *p* < 0.01, *** *p* < 0.001) is used for diet impact (HR_2HFD vs. HR_CD) and purple (* *p* < 0.05) is used for microbiota impact (NAFLR_2HFD vs. HR_2HFD) statistical comparisons; (**h**) AUC of the OGTT at D70; (**i**) non-fasting insulinemia at D90. HR, healthy human microbiota receiver mice; CD, control diet; 2HFD, high-fructose, high-fat diet; NAFLR, NAFL patient microbiota receiver mice; light green, HR_CD, HR mice on CD; dark green, HR_2HFD, HR mice on 2HFD; purple, NAFLR_2HFD, NAFLR mice on 2HFD; OGTT, oral glucose tolerance test; HOMA-IR, homeostatic model assessment of insulin resistance; AUC, area under the curve. D70, day 70, 7 weeks of 2HFD treatment; D90, day 90, 10 weeks of 2HFD treatment.

Seven weeks after FMT, on day 70, fasting insulinemia and glycemia were measured, and then an OGTT was performed. There were no significant differences between the HR_CD (light green bars) and HR_2HFD (dark green bars) groups in fasting insulinemia, glycemia and HOMA-IR (Figure 4a–c), indicating no effect of the diet on these parameters.

However, HR_2HFD mice had higher insulinemia, higher glycemia and higher insulin resistance 30 min after glucose compared to HR_CD controls, (38.5 ± 7.4 vs. 17.8 ± 3.0 mU/L, *p* = 0.019, 19.3 ± 1.1 vs. 13.9 ± 0.6 mmol/L, *p* < 0.001, HOMA-IR of 34.4 ± 7.6 vs. 10.9 ± 1.8, *p* = 0.009, respectively), showing a real impact of the 2HFD regimen on mice metabolism (Figure 4d–f). Moreover, compared to HR_CD controls, HR_2HFD mice showed higher blood glucose 15, 30 and 60 min after the glucose gavage (green stars, Figure 4g) and a higher AUC of OGTT (711± 35.4 vs. 545.5 ± 15.4 mg/dL.min, respectively, *p* < 0.001, Figure 4h), indicating lower glucose tolerance in HR_2HFD mice. At the end of the experiment, day 90, insulinemia was still higher in non-fasting HR_2HFD mice than in HR_CD (117.9 ± 24.3 vs. 41.6 ± 6.1 mU/L, respectively, *p* = 0.008, Figure 4i).

**Figure 5:**
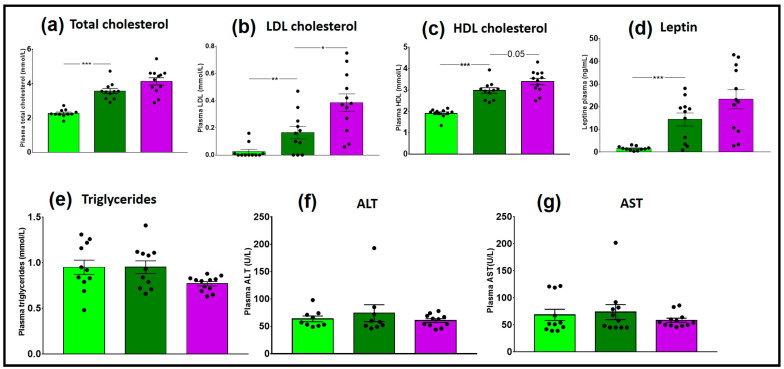
Plasma assay of CD- and 2HFD-fed HR vs. NAFLR mice, *n* = 11–12 mice/group. (**a**) Total cholesterol; (**b**) low-density lipoprotein (LDL) cholesterol; (**c**) high-density lipoprotein (HDL) cholesterol; (**d**) leptin; (**e**) triglycerides; (**f**) ALT; (**g**) AST. Light green, HR_CD, HR mouse on CD; dark green, HR_2HFD, HR mouse on 2HFD; purple, NAFLR_2HFD, NAFLR mice on 2HFD; LDL, low-density lipoprotein; HDL, high-density lipoprotein; ALT, alanine transaminase; AST, aspartate transaminase; HR, healthy human microbiota receiver mice; CD, control diet; 2HFD, high-fructose, high-fat diet; NAFLR, NAFL patient microbiota receiver mice; (* *p* < 0.05, ** *p* < 0.01, *** *p <* 0.001) were used for statistical comparisons.

HR_2HFD mice had also higher total cholesterol compared to HR_CD controls (3.7 ± 0.1 vs. 2.3 ± 0.1 mmol/L, *p* < 0.001), higher LDL cholesterol (0.16 ± 0.05 vs. 0.03 ± 0.02 mmol/L, *p* = 0.009) and higher HDL cholesterol (3.00 ± 0.13 vs. 1.90 ± 0.06 mmol/L, *p* < 0.001), indicating a real impact of the 2HFD regimen on HR_2HFD cholesterol metabolism (Figure 5a–c). Higher leptin levels were also observed in HR_2HFD mice (14.4 ± 2.9 vs. 1.6 ± 0.3 ng/mL, *p* < 0.001, Figure 5d). There were no significant differences between the HR_CD and HR_2HFD groups in plasma triglycerides, alanine transaminase (ALT) and aspartate transaminase (AST) (Figure 5e–g).

#### 3.2.3. Steatosis

**Figure 6:**
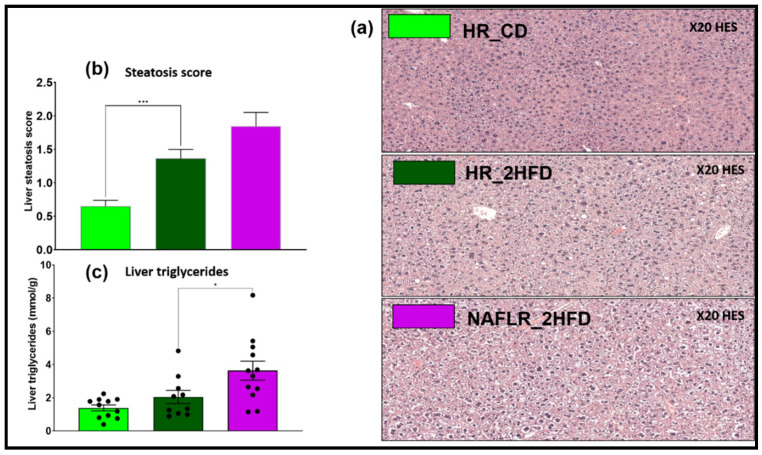
Liver steatosis analysis of CD- and 2HFD-fed HR vs. NAFLR mice, *n* = 10–12 mice/group. (**a**) Liver hematoxylin-eosin staining (HES) histology observation, magnification: 20×; (**b**) steatosis histological scores; (**c**) liver triglycerides. HR, healthy human microbiota receiver mice; CD, control diet; 2HFD, high-fructose, high-fat diet; NAFLR, NAFL patient microbiota receiver mice; light green, HR_CD, HR mice on CD; dark green, HR_2HFD, HR mice on 2HFD; purple, NAFLR_2HFD, NAFLR mice on 2HFD; HES, hematoxylin-eosin staining; (* *p* < 0.05, *** *p* < 0.001) were used for statistical comparisons.

HR_2HFD mice had a higher steatosis histological score compared to HR_CD controls at day 90 (1.4 ± 0.1 vs. 0.6 ± 0.1, respectively, *p* < 0.001, Figure 6a,b). However, non-significant differences were found for triglycerides concentrations in the liver (Figure 6c).

#### 3.2.4. Gene Expression

**Figure 7:**
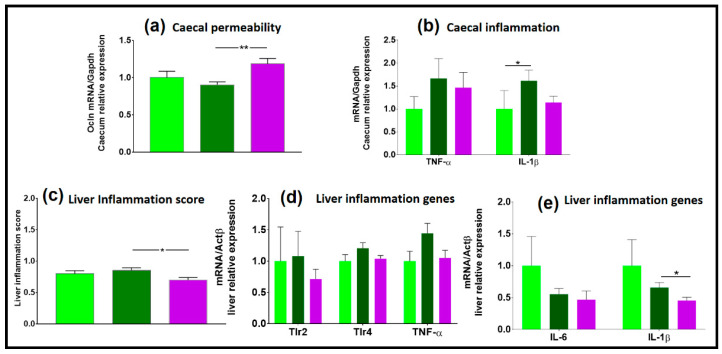
Gene expression in the caecum and in the liver, *n* = 12 mice/group. (**a**) Caecal permeability; (**b**) caecal inflammation; (**c**) liver inflammation score; (**d**,**e**) liver genes involved in inflammation. HR, healthy human microbiota receiver mice; CD, control diet; 2HFD, high-fructose, high-fat diet; NAFLR, NAFL patient microbiota receiver mice; light green, HR_CD, HR mice on CD; dark green, HR_2HFD, HR mice on 2HFD; purple, NAFLR_2HFD, NAFLR mice on 2HFD; (* *p* < 0.05, ** *p* < 0.01) were used for statistical comparisons.

The expression of occludin, a marker of gut permeability, did not differ between the two types of diet (Figure 7a) and there was no impact of the diet on genes involved in inflammation in the caecum (Figure 7b). There were no significant differences between the HR_CD and HR_2HFD groups in the expression level of genes involved in liver inflammation (Figure 7c–e).

TLR4 was overexpressed in HR_2HFD as compared with HR_CD (1.9 ± 0.2- vs. 1.0 ± 0.1-fold, respectively, *p* < 0.001), suggesting that it might be an important target of the diet (Figure S6b). Expression of TLR2 was not different in HR_CD and HR_2HFD (not shown). In 2HFD mice, the glucose transporter 5 (Glut5, 0.38- vs. 0.90-fold, respectively, *p* = 0.04) and ketohexokinase (KhK, 0.40 ± 0.04- vs. 1.00 ± 0.10-fold, respectively, *p* < 0.001) genes involved in carbohydrate homeostasis were underexpressed, suggesting a direct impact of the diet on carbohydrate absorption in the caecum (Figure S6c).

#### 3.2.5. SCFA Concentrations

There were no significant differences between the HR_CD and HR_2HFD groups in total caecal short-chain fatty acids (SCFA, Figure S6d). Compared to CD, caecal butyrate concentration was lower under the 2HFD regimen (1.50 ± 0.30 vs. 6.30 ± 0.93 µmol/g caecum contents, respectively, *p* < 0.001, Figure S6e). Total caecal BCFA concentration was 2-fold higher in HR_2HFD as compared with HR_CD (0.66 ± 0.09 vs. 0.33 ± 0.05 µmol/g caecum contents, respectively, *p* = 0.003), suggesting that it might be an important target of the diet (Figure S6f). In particular, isovalerate was higher under 2HFD conditions (Figure S6g).

#### 3.2.6. Microbiome

On day 6, at the basal state, the proportions of the different bacteria families were similar and characteristic of the C57BL/6J mice microbiota between the future HR_CD and HR_2HFD groups (Figure S4 and Figure 10). One week after inoculation, the most abundant families included *Prevotellaceae*, *Bacteroidaceae* and *Ruminococcaceae* in HR_CD. In contrast, *Prevotellaceae* were absent and replaced by a higher proportion of *Bacteroidaceae* in HR_2HFD (Figure S4). At the family level, there was an equivalent proportion of *Bacteroidaceae* and *Lachnospiraceae* between the two groups of mice on day 55, day 84 and day 90. *Prevotellaceae* and *Burkholderiaceae* were absent, and *Tannerellaceae* was less present in the HR_2HFD group, replaced by a higher proportion of *Ruminococcaceae*, *Desulfovibrionaceae* and other non-dominant families (Figure S4). All throughout the experiment, *Prevotellaceae* were present only under the CD regimen, probably due to a greater presence of fiber in the CD regimen. In both groups, the abundance of *Lachnospiraceae* increased with time and replaced, in abundance, the proportion of *Bacteroidaceae* (Figure S4). These observations are paired with a less diverse microbiota, lower proportion of *Lachnospiraceae*, absence of *Prevotellaceae* and high abundance of *Peptostreptococcaceae*, *Family XIII* and *Bacteroides* under the 2HFD regimen (Figure 11b).

Altogether, these observations indicated the development of early-phase NAFLD in mice after a 10 weeks’ 2HFD regimen, including increased body weight, adipose tissues, steatosis, cholesterolemia and glucose intolerance linked with the absence of *Prevotellaceae* and the gradual replacement of *Bacteroidaceae* by *Lachnospiraceae* as compared to mice fed the control diet.

### 3.3. Impacts of NAFL Human Fecal Microbiota (HR_2HFD vs. NAFLR_2HFD)

In this part, we compare the mice remaining on the same diet rich in fructose and fat for 10 weeks (2HFD) but receiving, for the first group, the healthy human fecal transplant (HR_2HFD) or, for the second group, the NAFL human fecal transplant (NAFLR_2HFD), in order to assess the effect of the NAFL human fecal microbiota.

#### 3.3.1. NAFL Human Fecal Microbiota Increased Body Weight Gain in Recipient Mice

Despite identical energy intake all throughout the experiment on the same 2HFD experimental regimen (Figure 3a), and compared to HR controls, NAFLR mice gained more weight after two weeks on 2HFD (with a mean increase difference in body weight of 8.0% at week 2, *p* = 0.003, and 16.1% at week 10, *p* < 0.001, Figure 3b). NAFLR_2HFD mice had a higher body weight than the HR_2HFD mice at the end of the experiment (39.5 g ± 1.9 vs. 35.0 g ± 0.8, respectively, *p* < 0.001). The only different condition between the two groups being the microbiota, it can be concluded that the microbiota from the overweight (BMI > 25) NAFL patient transferred this phenotype to the NAFLR mice.

#### 3.3.2. Plasma Assays

We investigated the impact of NAFL FMT on diet-associated metabolic dysfunction. Fasting insulinemia and glycemia were measured three weeks before euthanasia, and then an OGTT was performed. Non-fasting glycemia (22.1 ± 1.2 vs. 19.3 ± 1.1 mmol/L, respectively, *p* = 0.11) and HOMA-IR (45.5 vs. 28.2, respectively, *p* = 0.17) tended to be higher in NAFLR mice (Figure 4e,f). Therefore, the tendency to insulin resistance may have been transferred to the NAFLR mice. In particular, HR mice have improved glucose tolerance, which was significant 1 h and 1 h 30 min after the glucose gavage, as compared with NAFLR mice (purple stars, *p* < 0.05, Figure 4g). These tendencies were not confirmed by measurement of the AUC of OGTT on day 70 (*p* = 0.12, Figure 4h) and by non-fasting insulinemia sensitivity at euthanasia (*p* = 0.71, Figure 4i). As expected at this early stage of the disease, there were no differences between NAFL and healthy human plasma transaminases ALT (33 vs. 16 U/L, both under the 33 U/L threshold for humans) and AST (25 vs. 15 U/L, both under the 32 U/L threshold for humans) (Table 1). There was also no difference between NAFLR (ALT: 60.4 ± 3.4 U/L and AST: 58.6 ± 3.8 U/L) and HR (ALT: 74.0 ± 15.4 U/L and AST: 73.5 ± 13.9 U/L) mice transaminases (Figure 5f,g). No significant effect on total cholesterolemia (Figure 5a) but higher LDL cholesterolemia (0.39 ± 0.06 vs. 0.16 ± 0.05 mmol/L, respectively, *p* = 0.01, Figure 5b) were observed in NAFLR mice as compared to HR mice. Moreover, trends towards more HDL cholesterolemia (3.4 ± 0.1 vs. 3.0 ± 0.1 mmol/L, respectively, *p* = 0.05, Figure 5c) and leptinemia and less triglyceridemia were also observed in these mice compared to HR mice (Figure 5d,e). Plasma triglycerides were under the normal human threshold (<2.26 mmol/L) both in NAFL (0.85 mmol/L) and in healthy (0.62 mmol/L) individuals. Mice plasma triglycerides, NAFLR_2HFD (0.77 ± 0.02 mmol/L) and HR_2HFD (0.95 ± 0.07 mmol/L) did not reiterate the difference observed in human donors (NAFL donor: 0.85 mmol/L, healthy donor: 0.62 mmol/L) (Figure 5e).

**Figure 8:**
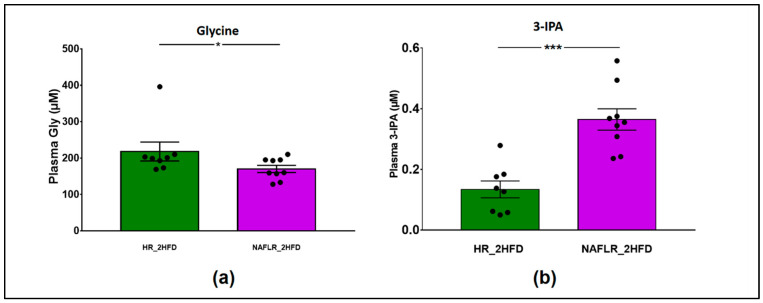
Metabolomic analysis of plasma in HR_2HFD and NAFLR_2HFD mice, *n* = 12 mice/group. (**a**) Plasma glycine (µM); (**b**) plasma 3-IPA (µM). 3-IPA, 3-Indolepropionic acid; HR, healthy human microbiota receiver mice; CD, control diet; 2HFD, high-fructose, high-fat diet; NAFLR, NAFL patient microbiota receiver mice; light green, HR_CD, HR mice on CD; dark green, HR_2HFD, HR mice on 2HFD; purple, NAFLR_2HFD, NAFLR mice on 2HFD; (* *p* < 0.05, *** *p* < 0.001) were used for statistical comparisons.

Regarding the metabolomic profile on 315 screened and validated metabolites, only glycine was more abundant in HR_2HFD mice than in the NAFLR_2HFD mice (200 vs. 160 µM, *p* < 0.05), and 3- Indolepropionic acid (3-IPA), an indole derivative, was more abundant in NAFLR_2HFD than in HR_2HFD (0.364 ± 0.035 vs. 0.134 ± 0.027 µM, *p* < 0.001) (Figure 8). The other metabolites were not discriminant between the two groups of mice.

#### 3.3.3. NAFL Human Fecal Microbiota Worsened Liver Steatosis in Mice

The NAFL patient liver was biopsied and steatosis was scored at 2.0 (Table 1). NAFLR_2HFD mice liver was harvested and steatosis was scored at 1.8 ± 0.2, whereas HR_2HFD steatosis was scored at 1.4 ± 0.1 Figure 6a). Histological observations and scorings of the liver sections revealed a tendency to store more lipids in NAFLR than in HR livers Figure 6b). This was confirmed by quantification of TG in the liver (3.6 ± 0.6 vs. 2.0 ± 0.4 mmol/g, respectively, *p* = 0.04, Figure 6c).

#### 3.3.4. NAFL Human Fecal Microbiota Transfer Modulated Liver Lipid Metabolism

**Figure 9:**
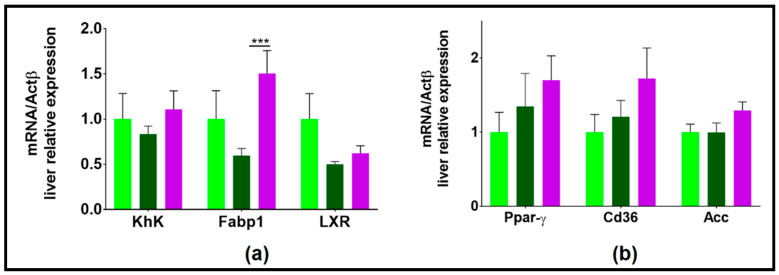
Hepatic expression of genes involved in lipid and carbohydrate metabolisms. (**a**) genes whose expression tended to decrease with 2HFD and then tended to increase with NAFL microbiota (**b**) genes whose expression tended to increase with 2HFD and even more with NAFL microbiota; HR, healthy human microbiota receiver mice; CD, control diet; 2HFD, high-fructose, high-fat diet; NAFLR, NAFL patient microbiota receiver mice; light green, HR_CD, HR mice on CD; dark green, HR_2HFD, HR mice on 2HFD; purple, NAFLR_2HFD, NAFLR mice on 2HFD; (*** *p* < 0.001) was used for statistical comparisons.

In the liver, the lipid transporter FABP1 (fatty acid binding protein 1) was overexpressed in NAFLR_2HFD as compared to HR_2HFD, suggesting an increased entry of free fatty acids (FFA) into mice hepatocytes (Figure 9a).

This is in agreement with a potential tendency to overexpress Ppar-γ, ACC and Cd36 in NAFLR_2HFD as compared to HR_2HFD (Figure 9b), which suggests increased lipogenesis in this group. Altogether, these results indicate that the NAFL human fecal microbiota transfer worsened liver steatosis in 2HFD-induced NAFLD mice through modulation of lipid metabolism.

#### 3.3.5. NAFL Human FMT Alleviated Inflammatory Markers in Liver and Caecum

Compared to healthy inocula, NAFL inocula induced an overexpression of occludin in the caecal mucosa (1.1- vs. 0.9-fold, respectively, *p* = 0.002, Figure 7a), a marker of the gut barrier, suggesting a lower caecal permeability for NAFLR mice. In parallel, the 2HFD regimen induced an increase in caecal expression of IL-1β, suggesting a caecal inflammation, and, as shown in the above part, that the transfer of the NAFL microbiota tended to reduce (1.1- vs. 1.6-fold, respectively, *p* = 0.09, Figure 7b). Liver inflammatory histological scores showed that there is less inflammation in NAFLR livers compared to HR livers (0.6- vs. 0.8-fold, respectively, *p* = 0.02, Figure 7c), which is associated with decreased expression of IL-1β in NAFLR livers (0.5- vs. 0.7-fold, respectively, *p* = 0.03, Figure 7e). This is in agreement with the trends observed on liver inflammatory markers. While 2HFD tended to increase TLR4 and TNF-α expression, as shown previously, the NAFL microbiota moderated this trend (1.0- vs. 1.2-fold, respectively, *p* = 0.09, for TLR4, and 1.0- vs. 1.4-fold, respectively, *p* = 0.06, for TNF-α, Figure 7d). Expression of TLR2 was similar in HR_2HFD and NAFLR_2HFD (*p* = 0.46, Figure 7d). These results suggest that NAFL human FMT may alleviate liver local inflammation at the early phase of 2HFD-induced NAFLD in mice.

#### 3.3.6. Caecum Weight and SCFA Concentrations

There were no significant differences between the HR_2HFD and NAFLR_2HFD groups in the caecum index (*p* = 0.45, Figure S6a), total caecal SCFA (*p* = 0.83, Figure S6d) and BCFA (*p* = 0.16, Figure S6f).

#### 3.3.7. Microbiome

**Figure 10:**
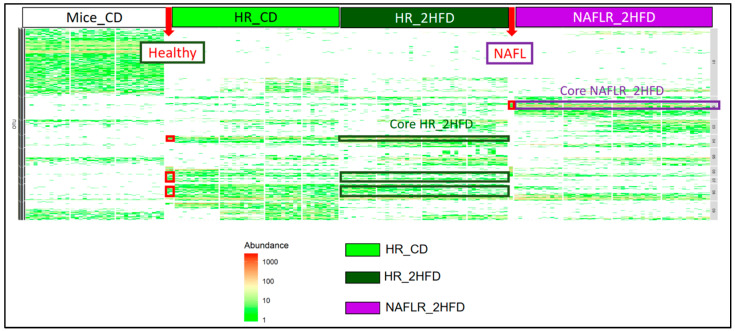
Heatmap characterization of differentially abundant and prevalent operational taxonomic units (OTUs) all throughout the experiment. CD, control diet; 2HFD, high-fructose, high-fat diet; Mice_CD, mice microbiota at basal state, day 6, on CD; Healthy, healthy human microbiota; NAFL, NAFL patient microbiota; HR, healthy human microbiota receiver mice; NAFLR, NAFL patient microbiota receiver mice; HR_CD, HR on CD regimen; HR_2HFD, HR on 2HFD regimen; NAFLR_2HFD, NAFLR on 2HFD regimen.

The proportion of the different bacterial families was similar at the basal state and characteristic of the C57BL/6J mice microbiota between the future HR_2HFD and NAFLR_2HFD groups (Figure 10, Figure S4). One week after inoculation, the most abundant families included *Bacteroidaceae* and *Ruminococcaceae* in HR_2HFD, while *Bacteroidaceae* were less abundant and replaced by a higher proportion of *Lachnospiraceae* in NAFLR_2HFD (Figure S4). On days 55 and 84, there was an equivalent proportion of *Bacteroidaceae, Lachnospiraceae* and *Ruminococcaceae* between the two groups (Figure S4). *Atopobiaceae* were more abundant, reducing the proportion of *Bacteroidaceae*, in NAFLR at days 84 and day 90 (Figure S4). At day 90, HR_2HFD mice showed a higher α-diversity (observed richness: 223.1 ± 5.1, Chao1: 268.1 ± 9.4, Figure 2a, Figure S3a) and therefore a significantly higher richness of OTUs than the NAFLR_2HFD mice (observed richness: 190.1 ± 4.3, Chao1: 227.9 ± 8.9, Figure 2a, Figure S3a). All throughout the experiment, the abundance of *Lachnospiraceae* increased with time in both groups and replaced, in abundance, the proportion of *Bacteroidaceae* (Figure S4). Surprisingly, *Atopobiaceae*, initially abundant in all mice microbiota, were totally depleted by antibiotics and returned to the initial level after 2 months in the NAFLR group only (Figure S4).

We characterized the more differentially abundant and prevalent OTUs between the NAFLR_2HFD and HR_2HFD microbiota, which were already more differentially abundant and prevalent OTUs between the NAFL and healthy *inocula* and not originally present in mice feces before antibiotic treatment (Mice_CD feces, Figure 10). These OTUs were, respectively, named Core HR_2HFD and Core NAFLR_2HFD. The differentially abundant OTUs between healthy, NAFL *inocula*, HR_2HFD and NAFLR_2HFD feces are represented in Supplementary Figure S7.

**Figure 11:**
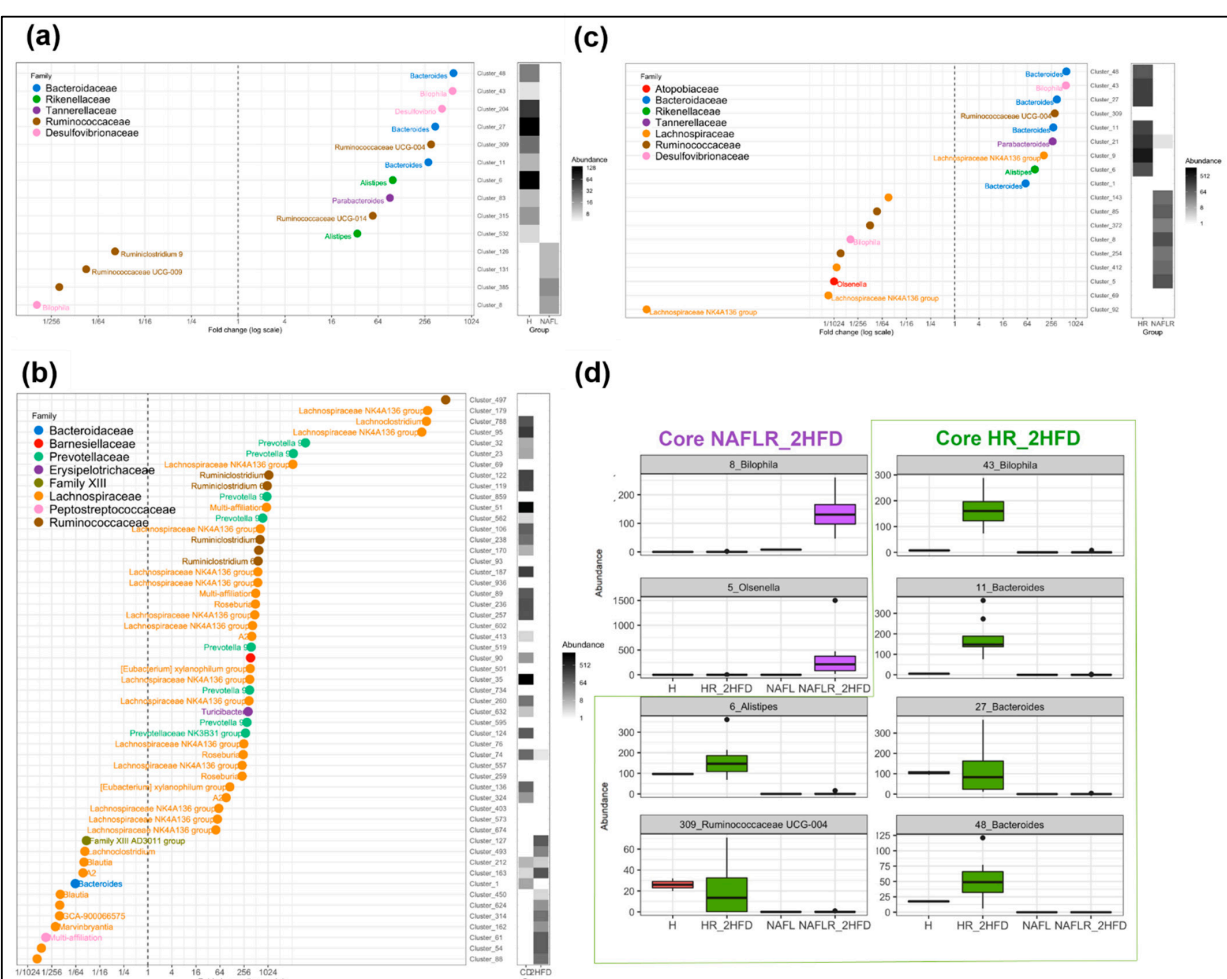
Graphical representation of differentially abundant OTUs, having a large fold change and significant effect size in addition to high relative abundance between (**a**) healthy, H, and NAFL *inocula*; (**b**) HR_CD and HR_2HFD groups on day 90; (**c**) HR_2HFD and NAFLR_2HFD groups on day 90. Each OTU is represented by a dot and colored according to its taxonomic classification at the family level. Taxonomy at the genus or species level is also indicated, when available, next to each OTU. A logarithmic scale (log-2) was used for the x axis. (**d**) Core NAFLR_2HFD and Core HR_2HFD abundance through the groups, corresponding, respectively, to the OTUs differentially abundant and transferred from *inocula* to mice. HR, healthy human microbiota receiver mice on 2HFD; NAFLR, NAFL patient microbiota receiver mice on 2HFD; 2HFD, high-fructose, high-fat diet.

Eighteen OTUs were found differentially abundant between the NAFLR_2HFD and HR_2HFD microbiota, 9 OTUs were more abundant in NAFLR_2HFD (left part, Figure 11c) and 9 OTUs were more abundant in HR_2HFD (right part, Figure 11c). *Lachnospiraceae* and *Ruminococcaceae* were more abundant in NAFLR_2HFD (left part, Figure 11c), while *Bacteroidaceae* was more abundant in HR_2HFD (right part, Figure 11c), and *Olsenella* (Cluster 5) specifically characterized the NAFLR mice microbiota. All these OTUs are framed in blue in Figure S7. *Alistipes* (Cluster 6), *Bacteroides* (Cluster 11), *Bacteroides* (Cluster 27), *Bacteroides* (Cluster 48), *Ruminococcaceae UCG-004* (Cluster 309) and *Bilophila* (Cluster 43) characterized the transfer from the healthy *inocula* to the HR_2HFD mice microbiota and correspond to Core HR_2HFD (Figure S7, Figure 11d). *Bilophila* (Cluster 8) characterized the transfer from the NAFL *inocula* to the NAFLR mice microbiota and corresponds to Core NAFLR_2HFD (Figure S7, Figure 11d).

## 4. Discussion

In the present manuscript, we looked for the causal contribution of the human intestinal microbiota as a determining factor in the early phases of NAFLD by transplanting the human microbiota to mice. Specific pathogen-free mice, pretreated with a mixture of antibiotics, were colonized with microbiota originating from an NAFL patient or a healthy individual. We validated by 16S and shotgun sequencing a partial time-limited human microbiota transfer to mice. We verified that our experimental high-fructose, high-fat diet (2HFD) condition alone allows the development of an early-phase NAFLD model in mice. We show that due to the feeding with 2HFD, mice were overweight and had more adipose tissues and liver steatosis and higher total blood cholesterol, leptinemia, glucose intolerance and insulin resistance. In addition, gut inflammation and permeability were slightly affected by the regimen. The 2HFD condition was also associated with a lower diversity of the microbiota and a lower butyrate caecal concentration, whereas the concentrations of BCFA such as isovalerate were doubled.

Compared to the healthy microbiota recipient mice, the transfer of the NAFL microbiota led to the development of a more obesogenic profile, including increased bod weight, higher liver steatosis and higher blood levels of cholesterol and TG in NAFL recipient mice. Unexpectedly, this was associated with improvements in some inflammatory markers in the liver and in gut barrier markers in the caecum. These observations were paired with a less diverse microbiota, dominated by *Lachnospiraceae* and *Ruminococcaceae* families, and a high abundance and prevalence of *Bilophila* and *Olsenella* in NAFLR mice.

In our study, we found that some dominant phyla such as Bacteroidetes, families such as *Ruminococcaceae* and genera such as *Bacteroides*, *Alistipes*, *Parabacteroides*, *Desulfovibrio* and *Bilophila* drive the ecological differences observed between the NAFL patient and the healthy individual. Both *inocula* from the healthy individual and the NAFL patient showed a lower α-diversity in comparison with the recipient mice, maybe because mice were not initially germ-free (GF). This could be considered as an unsatisfactory bias to the study but has the merit of being as close as possible to the clinical practices of FMT [13]. We observed a lower abundance of the Bacteroidetes phylum in the NAFL patient compared to the healthy individual as described in the literature [6], and this difference was also observed between the HR_2HFD and NAFLR_2HFD groups. On the contrary of what can be observed in other studies [6], we observed that *Ruminococcaceae* were more abundant and *Escherichia-Shigella* were less present in our NAFL patient than in our healthy individual. We especially did not detect *Lactobacillaceae*, *Veillonellaceae* or *Dorea* which have been recently shown to correlate with health and disease markers [36]. The more abundant and prevalent OTUs in the NAFL *inocula* were *Ruminococcaceae UCG-009*, *Bilophila* and *Ruminiclostridium 9*. The latter has been recently found in high-fat diet-fed mice receiving resveratrol [37], and this abundance increased in overweight people eating whole grain wheat [38]. *Ruminococcaceae UCG-009* was linked with induced inflammation [39] and hyperlipidemia in mice [40,41], while *Bilophila* is well known to aggravate high-fat diet-induced metabolic dysfunctions in mice [42]. The ten OTUs more abundant and prevalent in the healthy *inocula* were *Ruminococcaceae UCG-004*, *Ruminococcaceae UCG-014*, *Bacteroides*, *Alistipes*, *Desulfovibrio*, *Bilophila* and *Parabacteroides*. In the literature, *Ruminococcaceae UCG-004* seems to be considered as a pathogenic taxon, whereas *Ruminococcaceae UCG-014* is considered as a beneficial one [43]. *Bacteroides ovatus* was found to exert protective effects against the development of metabolic disorders in mice [14]. In their attempt to correlate gut microbiome composition with health and disease markers, Manor et al.’s results suggest that *Ruminococcaceae* and *Bacteroides* associations with host factors might result indirectly from the correlations between these genera and diversity, so they do not have to be considered on their own [36]. In terms of pathogenicity, there is contrasting evidence indicating that *Alistipes* may have protective effects against some diseases, including liver fibrosis, colitis, cancer immunotherapy and cardiovascular disease. In contrast, other studies indicate that *Alistipes* is pathogenic in colorectal cancer and is associated with mental signs of depression [44]. *Desulfovibrio* is an endotoxin producer, so it is usually described as pathogenic in the literature [45]. *Parabacteroides* alleviates obesity and metabolic dysfunctions in mice [46] and has a functional GABA-producing pathway [47].

We also verified that our experimental 2HFD regimen alone allows the development of early-phase NAFLD in mice by comparing phenotypes of mice inoculated with the same healthy *inocula* fed a control diet (CD) or high-fructose, high-fat diet (2HFD). We show that due to the feeding with 2HFD for ten weeks, mice gained more body weight, more adipose tissues weight, higher steatosis histological scores and higher total circulating cholesterol, leptin levels and glucose intolerance, but no differences in plasmatic triglycerides (TG), ALT or AST were observed. We also show that HR_2HFD had a lower caecum index than HR_CD with an overexpression of TLR4 and IL-1β genes, suggesting that the gut inflammation and permeability might be affected by the diet. Our results are consistent with those of Kawabata et al., 2019, where a high-fructose diet induced epithelial barrier dysfunction, including reduced expression of the tight junction protein occludin and increased inflammatory markers [48]. We also observed an overexpression of the TLR4 gene in the caecum, enforcing the argument that the 2HFD-induced change in the gut microbiota exacerbates inflammation and obesity in mice via the TLR4 signaling pathway as described previously [49]. The decrease in the caecum index may be explained by a reduced total caecal content due to high-fat diet feeding, involving a decrease in the bacteria diversity as previously described [50]. 2HFD lowered the butyrate caecal concentration and doubled BCFA such as isovalerate, which may contribute to the effects on host physiology. Indeed, a number of in vivo studies using rodent model showed that supplementation with butyrate reduced hepatic fat accumulation, decreased hepatic inflammation and suppressed cholesterol synthesis [51,52,53], and a reduction in butyrate amounts following a high-fat, high-fructose diet has been previously described [54]. Caecal BCFA have already been associated with hepatic TG [55] and the gut microbiota-produced isovalerate has been found higher in mice developing NAFLD [14]. These compounds have been considered as being detrimental for colonic and metabolic health [51,56,57,58].

These observations were paired with a less diverse microbiota, lower proportion of *Lachnospiraceae*, absence of *Prevotellaceae* and high abundance of *Peptostreptococcaceae*, *Family XIII* and *Bacteroides* with the 2HFD regimen. As in the study from Zeng et al., 2020 [59], the 2HFD microbiota was enriched with the *Lachnospiraceae* family, a secondary bile acid producer. *Peptostreptococcaceae*, a decarboxylating amino acid family [51], has been found in the human liver [60] and was associated with a high-caloric diet in mice [61]. *Family XIII* is known to increase in a high-fat diet [62] and is associated with inflammation [63].

Our data also show specific impacts of the NAFL human fecal microbiota during this 2HFD induction. The NAFL human fecal microbiota increased mice body weight and circulating LDL cholesterol in recipient mice, but not plasma TG and glucose intolerance. Our results are consistent with those of Ridaura et al. (2013), who transferred obesity through FMT from human obese twin donors to GF mice [64]. By contrast, Chiu et al. (2017) did not observe differences in the evolution of the body weight of mice transplanted with healthy normal BMI individuals’ or NASH overweight patients’ microbiota under CD or HFD. This discrepancy could be explained because they transferred pooled human fecal microbiota of 10 healthy individuals to GF mice on one hand and pooled human fecal microbiota of 10 NASH liver biopsied patients to GF mice on the other hand [65]. Here, NAFL human FMT also worsened liver steatosis in mice similarly to our previous results using mice-to-mice microbiota transplant [14]. These findings confirm that the gut microbiota markedly impacts the lipid metabolism and steatosis in the liver and that NAFL may be transmissible by gut microbiota transplantation. Whether this result is confirmed by FMT from human to human still needs to be determined and would have a huge impact on the selection of future FMT donors in clinical practice. Our results also complemented those of Hoyles et al. (2018), who independently transferred the fecal microbiota from three NAFL patients (grade 3, >66% steatosis) to eight antibiotic-treated mice (neomycin + ampicillin + metronidazole) in parallel with fecal microbiota transfer from three healthy individuals (grade 0, <5% steatosis) [12]. The authors noted a transfer of steatotic and metabolic phenotypes and demonstrated that the fecal microbiota obtained from patients with steatosis initiated hepatic lipid accumulation and affected the phenome of recipient mice through FMT in 2 weeks without HFD, reinforcing the causal role of the microbiota in steatosis. Not only did the donor microbiota from patients with steatosis trigger hepatic TG accumulation in recipient mice but it also affected the hepatic transcriptome, through an increased expression of genes involved in lipid metabolism such as FABP1. Another study using FMT from human to GF mice showed that the baseline (pre-intervention) gut microbiota from one genetically obese child with Prader–Willi syndrome induced liver steatosis in GF mice fed a normal diet, indicating that the gut microbiota from a genetically obese human could promote the onset of liver steatosis in mice independently from diet and genetic factors. This microbiota was characterized by a high abundance of *Bacteroides*, *Parabacteroides*, *Ruminococcus* and *Bilophila* [66]. These taxa are regarded as potential pathogens on account of their genetic potential to produce toxic co-metabolites such as trimethylamine N-oxide and indoxyl sulfate [67,68], and *Bilophila* has been linked with metabolic and inflammatory diseases [42,69]. They noted that the sole hepatic gene expression to be consistently modified all throughout the duration of the experiment was that of FABP1, involved in lipid binding and transport [66]. Interestingly, FABP1 was overexpressed in our study in NAFLR mice, suggesting similar mechanisms linking the microbiota to steatosis in the two studies. In agreement with our results suggesting a reinforcement of tight junctions via occludin expression, Zhou et al. (2017) showed that ZO-1 expression is increased after mice-to-mice FMT, thus modulating the intestinal permeability [70]. Development of early diet-induced hepatic steatosis in mice partly results from alterations in intestinal barrier function. Furthermore, protective effects of antibiotic treatment on the early signs of hepatic steatosis were associated with a protection against the loss of the tight junction protein ZO-1 in the small intestine. These data further support the hypothesis that changes in the intestinal microbiota might be critical in the development of intestinal barrier dysfunction in patients with NAFLD [71].

In our study, FMT did not affect caecum weight and SCFA caecal concentrations, suggesting that the mechanism involved in the phenotypic observations may not involve SCFA. Conversely, plasmatic glycine was less abundant and 3-IPA was more abundant in NAFLR_2HFD mice. In a recent study, the bacterial metabolism of BCAA and aromatic amino acids has also been implicated in NAFL and derived microbial metabolites including 3-(4-hydroxyphenyl)lactate and phenylacetic acid have been associated with steatosis and fibrosis development [11,12]. Phenylalanine metabolism has also been recently linked to inflammation-associated mitochondrial dysfunction as a potential mechanism for acute chronic liver failure (ACLF), a later consequence of NAFLD in decompensated cirrhosis [72]. In our study, another link between these metabolisms must be underlined through the increase in 3-indolepropionic acid (3-IPA) in NAFLR mice. This metabolite derives from the tryptophan metabolism by the gut bacteria which has been shown activated in ACLF [72]. *Bacteroides*, *Eubacterium* and *Clostridium* are well-known bacteria involved in proteolytic fermentation, producing indole from tryptophan. The indole derivatives, including 3-IPA, have been shown to decrease gut inflammation and prevent gut barrier dysfunction through aryl hydrocarbon receptor (AhR) activation [51,73,74]. Therefore, 3-IPA production by the gut microbiota may be involved in the reduction in inflammatory and permeability profiles in NAFLR mice. Interestingly, the implication of AhR in diet-induced obesity and NAFLD has also been described [75,76,77,78].

We also found a less diverse microbiota, higher proportion of some species from *Lachnospiraceae* and *Ruminococcaceae* and high abundance of *Bilophila* and *Olsenella* in NAFLR_2HFD mice. In the literature, the fecal microbiome of patients with steatosis has been reported to harbor bacterial strains normally living in the small intestine and oral cavity [12,79]. Accordingly, *Olsenella*, that we found specifically associated with the NAFLR mice, is well-detected in the subgingival microbiota and may be implicated in periodontal disease [80]. Moreover, patients with steatosis had fewer butyrate-producing *Lachnospiraceae* and *Ruminococcaceae*, in comparison with healthy individuals. In our study, we observed no difference in the total abundance of *Lachnospiraceae* and *Ruminococcaceae*, which may be responsible for the absence of difference observed in butyrate production, but specific abundant and prevalent OTUs from *Lachnospiraceae* and *Ruminococcaceae* were found higher in NAFLR mice.

In conclusion, we transplanted the microbiota from a patient with fatty liver and from a healthy individual to two groups of mice and showed that the microbiota composition in recipient mice resembled the microbiota composition of their respective human donor. Following administration of a high-fructose, high-fat diet, mice that received the human NAFL microbiota developed features of NAFLD including increased body weight, steatosis and plasma cholesterol. We identified bacterial genera and OTUs that were associated and therefore potentially responsible for the different phenotypes observed.

Our study displays several limitations; therefore, the results must be interpreted with caution. First, despite the fact we tried to match donors for a maximum of potential confounding factors, a few (including race and smoking habits) were different between the selected donors and it is not clear how this could have impacted our results. Further, we transplanted the gut microbiota from two donors only and we can only speculate whether similar results would be obtained using the microbiota from different healthy and NAFL donors. While transplantation of the human microbiota to mice has been considered as a relevant model to prove causality and decipher the role of the gut microbiota in health and disease, the inability of certain human bacterial species to colonize recipient mice guts may also constitute a limitation of this approach. Finally, only male mice were used in our study to avoid bias due to hormone variation in females, and therefore we cannot affirm that similar conclusions would be drawn in female mice. Although the mechanisms of interaction between specific gut microbes and the host metabolism still need further exploration, our results confirm that the gut bacteria play a role in obesity and steatosis development and that targeting the gut microbiota may be a preventive or therapeutic strategy in NAFLD management.

## Figures and Tables

**Table 1:** Clinical design and selection. (a) Diagnostic data and (b) biological data for the non-alcoholic fatty liver (NAFL) patient and the healthy control.

Diagnostic	Threshold Values	Healthy Human	NAFL Human
Age (years)		63	71
Female		yes	yes
White		yes	yes
Hispanic or Latin		no	yes
Smoker		actual	never
Duration of tobacco consumption (years)		30	0
Quantity consumed (packs of cigarettes/day)		0.25	0
Duration since stop date (years)		0	0
Alcohol consumption (glasses)		0	0
Body weight (kg)		60	72
Waist circumference (cm)	<80	75	96
Height (cm)		168	160
BMI	<25	21 (normal)	28 (overweight)
Diet		omnivorous	omnivorous
Liver steatosis score (% steatosis)	<5%	-	2 (60%)
**Biological Data**	**Threshold Values**	**Healthy** **Human**	**NAFL Human**
Haptoglobuline (g/L)		1.3	1.27
Alpha2-macroglobulin (g/L)		1.67	1.54
Ferritine (µg/L)		65	202
Bilirubine (µmol/L)		3.2	11
Hemoglobin A1c (%)		5.6	5.7
Fasting blood glucose (mmol/L)		5.29	5.6
Fasting plasma insulin (mU/L)		6.6	8
HOMA-IR	<2	1.55	1.99
Plasma ALT (IU/L)	<33	16	33
Plasma AST (IU/L)	<32	15	25
Plasma triglycerides (mmol/L)	<2.26	0.62	0.85

## Data Availability

Not Applicable.

## Supplementary Materials

The following are available online at https://www.mdpi.com/2076-2607/9/1/199/s1, Figure S1: Initial mice random repartition per group. No brothers between mice, 3 random iterations needed to balance the dataset between initial mice body weight (g) represented as mean+ S.E.M. after randomization in three groups at day 20, *n* = 12 mice per group (a) and the 9 cages at the provider, represented by colors (b). Figure S2: *Inocula* viability by FACS after thawing. (a) Healthy *inocula*; (b) NAFL *inocula*. Figure S3: 16S *inocula* and caecal microbiome: (a) α-diversity through different ecological indexes; (b) family composition with the 12 more abundant families; (c) genus composition with the 14 more abundant genera. Figure S4: 16S fecal and caecal mice microbiome relative abundance at family level and different timepoints. Figure S5: Other organs measurements. (a) Mesenteric fat/100g mouse; (b) epididymal fat/100g mouse; and (c) liver index (%) = liver weight (g)/mice final body weight (g) ×100. Figure S6: SCFA, BCFA modulation and caecal genes expression in 10 weeks’ 2HFD-induced NAFLD mice, *n* = 9–10 mice/group. (a) Caecum index (%) = caecum weight (g)/mice final body weight (g) ×100; (b) caecal TLR4 gene expression; (c) caecal carbohydrate homeostasis genes expression (Glut5, KhK); (d) total caecal SCFA in µmol/g caecum content; (e) caecal butyrate in µmol/g caecum content; (f) total caecal BCFA in µmol/g caecum content; (g) caecal isovalerate in µmol/g caecum content. TLR4, Toll-like receptor 4; Glut5, glucose transporter 5 gene; KhK, ketohexokinase gene; SCFA, short-chain fatty acids; BCFA, branched chain fatty acids. Figure S7: Abundance heatmap representation of differentially abundant OTUs (DAOTUs) between healthy H *inocula*, NAFL *inocula*, HR_2HFD and NAFLR_2HFD groups on day 90, having a large fold change and significant effect size in addition to high relative abundance. Each OTU is a row and represented according to its taxonomic classification at the family level. OTUs on Figure 11, framed in blue. OTUs corresponding to the effect of 2HFD diet framed in red. HR, healthy human microbiota receiver mice on 2HFD; NAFLR, NAFL patient microbiota receiver mice on 2HFD; 2HFD, high-fructose, high-fat diet. Table S1: Mice HFD vs. CD.
