# Supplementary material for: Fecal Microbiota Transplant from Human to Mice Gives Insights into the Role of the Gut Microbiota in Non-Alcoholic Fatty Liver Disease (NAFLD)
Source: Microorganisms. 2021 Jan 19;9(1):199. doi: 10.3390/microorganisms9010199 (PMC7833443; doi:10.3390/microorganisms9010199)
Supplement: Supplementary file 1 [file microorganisms-09-00199-s001.pdf]

microorganisms

Supplementary Materials: Fecal Microbiota Transplant from Human to Mice Gives Insights into the Role of the Gut Microbiota in Non-Alcoholic Fatty Liver Disease (NAFLD)

Table 1. Mice [1] CD vs. [2] HFD.

Diet composition	[1] CD %	[2] HFD %
Crude protein	21.4	26.2
Crude fat	5.1	34.9
Crude fiber (cellulose)	4.0	6.5
Crude ash	5.4	3.2
N free extracts (carbohydrates)	52.0	26.3
Starch	33.6	0
Sugar	4.4	8.9
Dextrin	3 ND	16.2
Metabolizable Energy (kcal/g)	3.4	5.2
Protein (%)	26	20
Fat (%)	14	60
Carbohydrates (%)	60	20

[1] CD, γ-irradiated 45 kGy mice conventional diet, SAFEA03 R03-40 diet (SAFE, Augy, France).
[2] HFD, γ-irradiated 45 kGy high-fat diet D12492 (Research Diets, Lynge, Denmark).
[3] ND, not determined.

Citation: Burz, S.D.; Monnoye, M.; Philippe, C.; Farin, W.; Ratziu, V.; Strozzi, F.; Paillarse, J.-M.; Chêne, L.; Blottière, H.M.; Gérard, P. Fecal Microbiota Transplant from Human to Mice Gives Insights into the Role of the Gut Microbiota in Non Alcoholic Fatty Liver Disease (NAFLD). *Microorganisms* **2021**, *9*, 199. https://doi.org/10.3390/microorganisms9010199

Received: 22 December 2020
Accepted: 12 January 2021
Published: 19 January 2021

Publisher's Note: MDPI stays neutral with regard to jurisdictional claims in published maps and institutional affiliations.

Copyright: © 2021 by the authors. Submitted for possible open access publication under the terms and conditions of the Creative Commons Attribution (CC BY) license (http://creativecommons.org/licenses/by/4.0/).

Figure S1 .

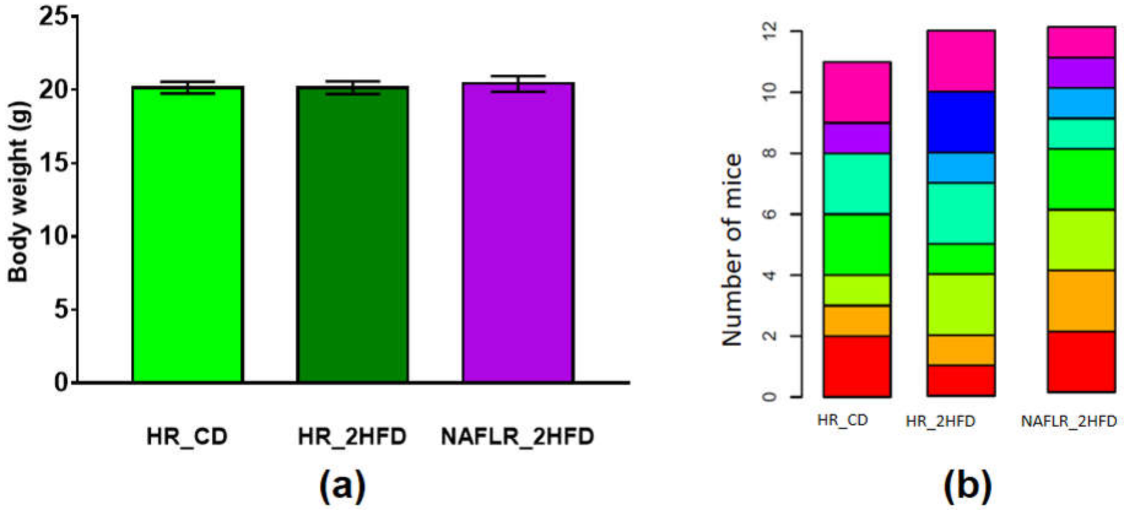

Figure S1. Initial mice random repartition per group. No brothers between mice, 3 random iterations needed to balance the dataset between initial mice body weight (g) represented as mean+ S.E.M. after randomization in three groups at day 20, n = 12 mice per group (**a**) and the 9 cages at the provider, represented by colors (**b**).

Figure S2 .

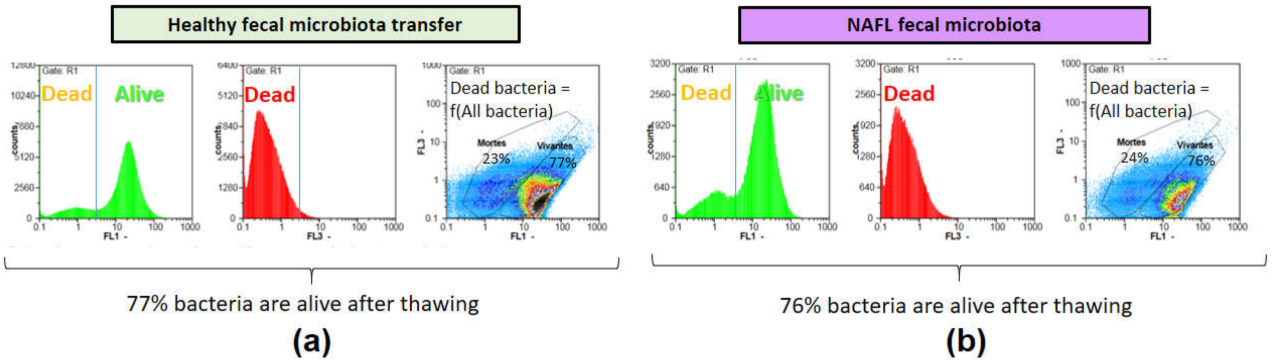

Figure S2. *Inocula* viability by FACS after thawing. (**a**) Healthy *inocula*; (**b**) NAFL *inocula*.

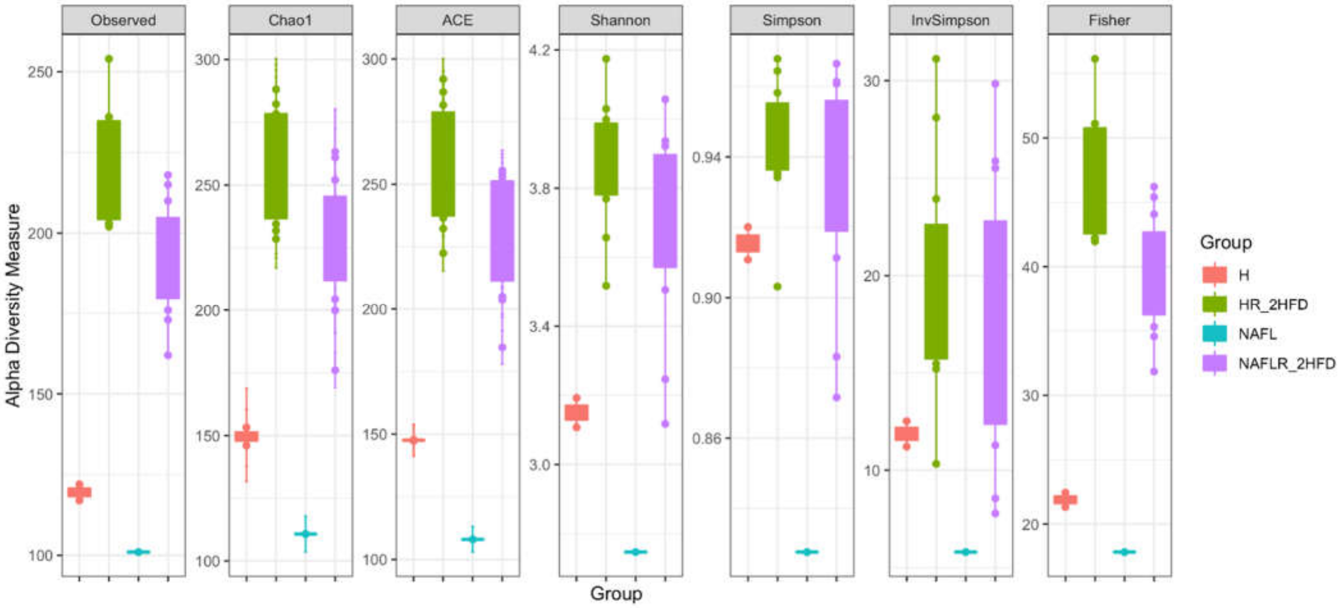

(**a**)

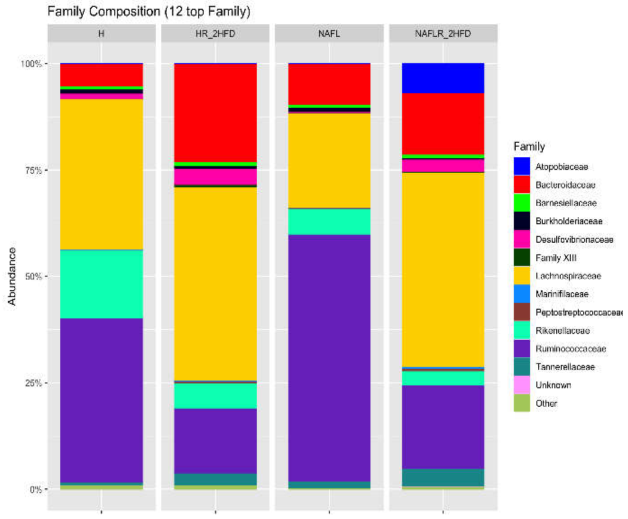

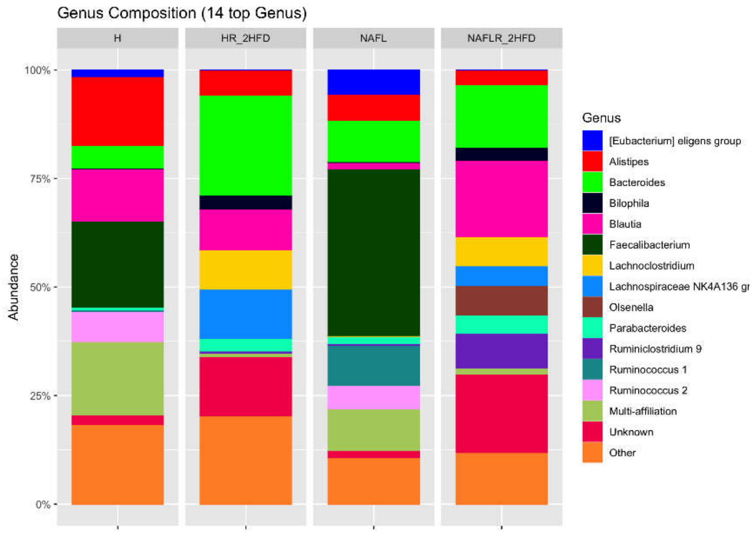

(**b**) (**c**)

Figure S3. Analysis of *inocula* and caecal microbiome: (**a**) α-diversity through different ecological indexes; (**b**) family composition with the 12 more abundant families; (**c**) genus composition with the 14 more abundant genera.

Figure S4.

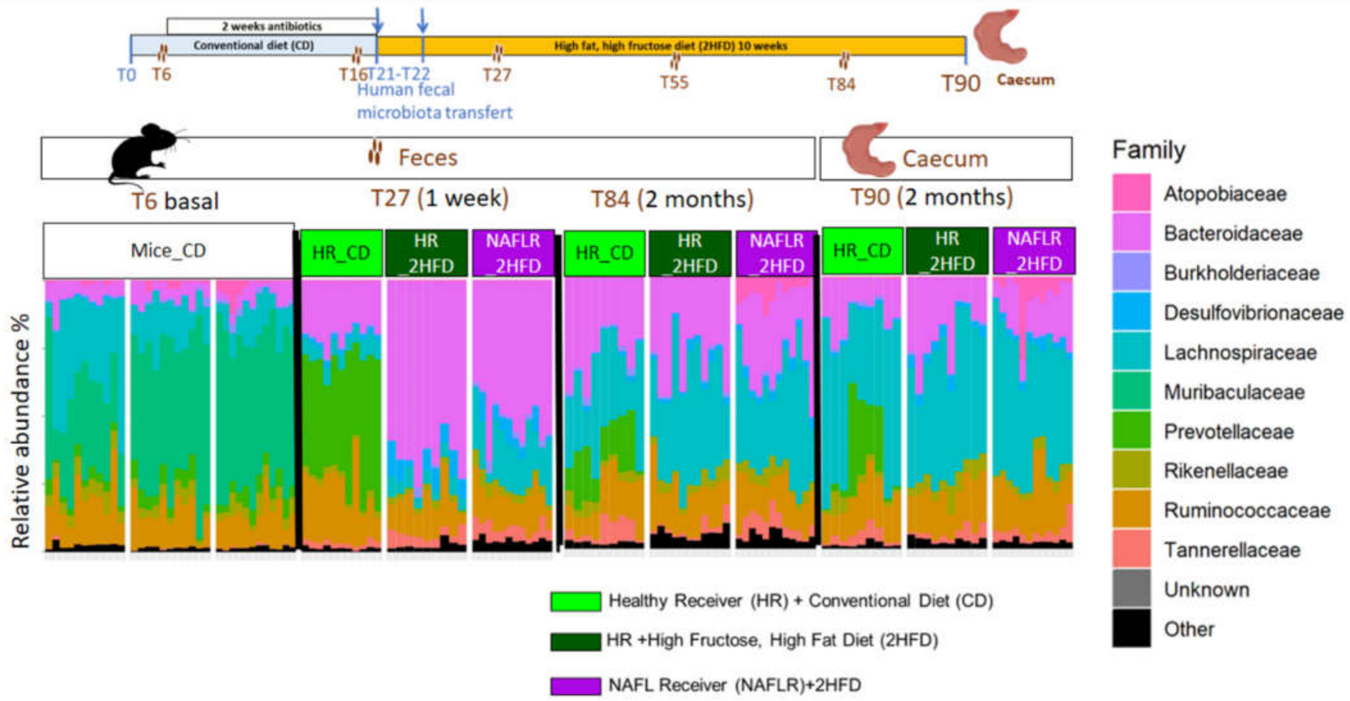

Figure S4. Analysis of fecal and caecal mice microbiome at the family level and different timepoints.

Figure S5.

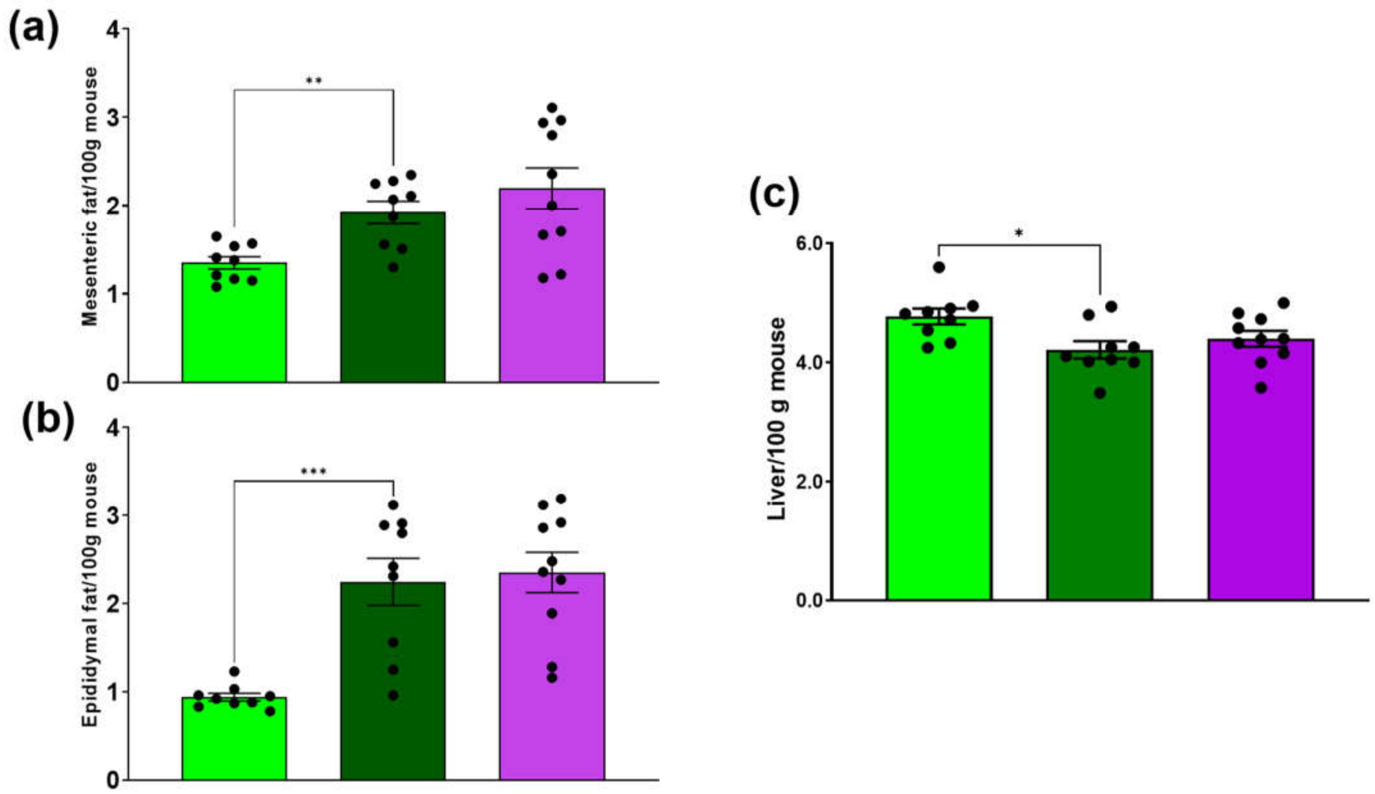

Figure S5. Weights of different organs (**a**) Mesenteric fat/100 g mouse; (**b**) epididymal fat/100 g mouse; and (**c**) liver index (%) = liver weight (g)/mice final body weight (g) ×100.

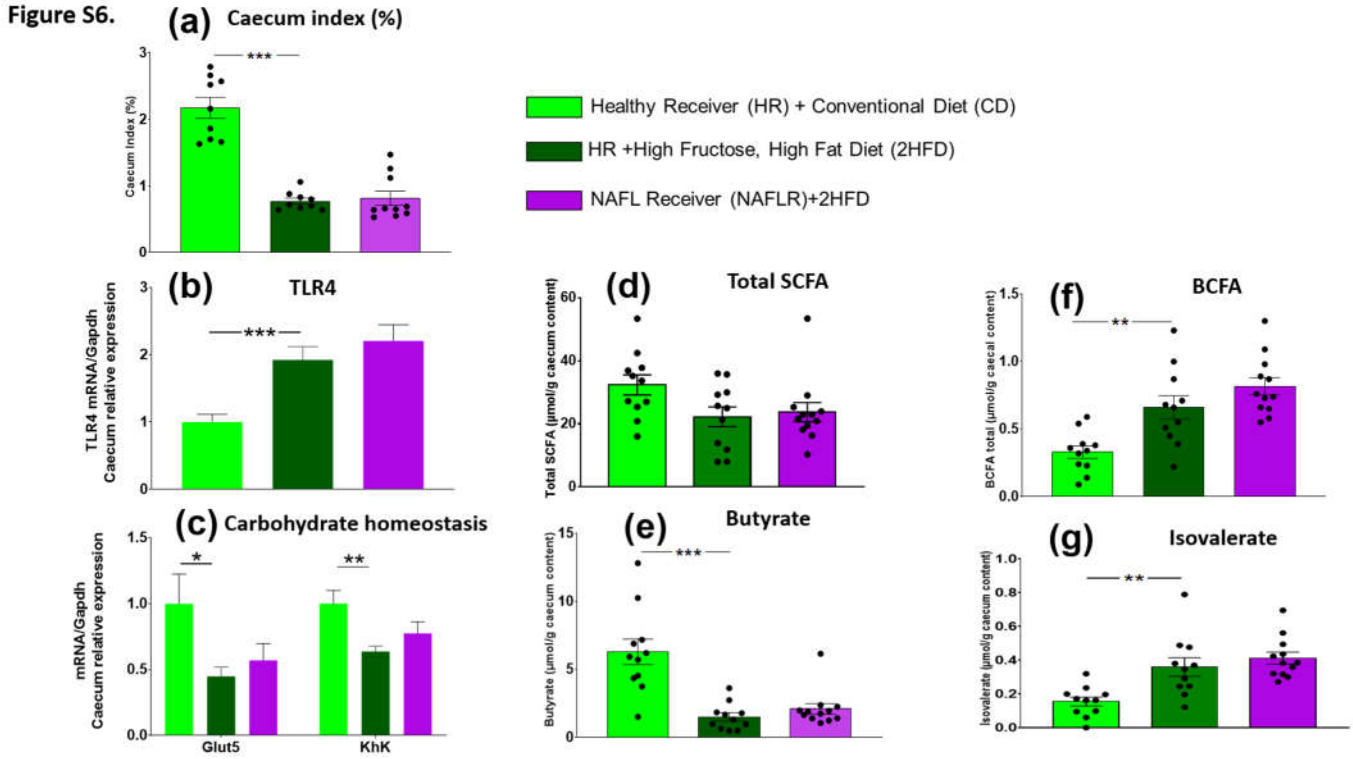

Figure S6. Caecal genes expression, and BCFA and SCFA concentrations in 10 weeks' 2HFD-induced NAFLD mice, *n* = 9–10 mice/group. (a) Caecum index (%) = caecum weight (g)/mice final body weight (g) ×100; (b) caecal TLR4 gene expression; (c) caecal carbohydrate homeostasis genes expression (Glut5, KhK); (d) total caecal SCFA in µmol/g caecum content; (e) caecal butyrate in µmol/g caecum content; (f) total caecal BCFA in µmol/g caecum content; (g) caecal isovalerate in µmol/g caecum content. TLR4, Toll-like receptor 4; Glut5, glucose transporter 5 gene; KhK, ketohexokinase gene; SCFA, short-chain fatty acids; BCFA, branched chain fatty acids.

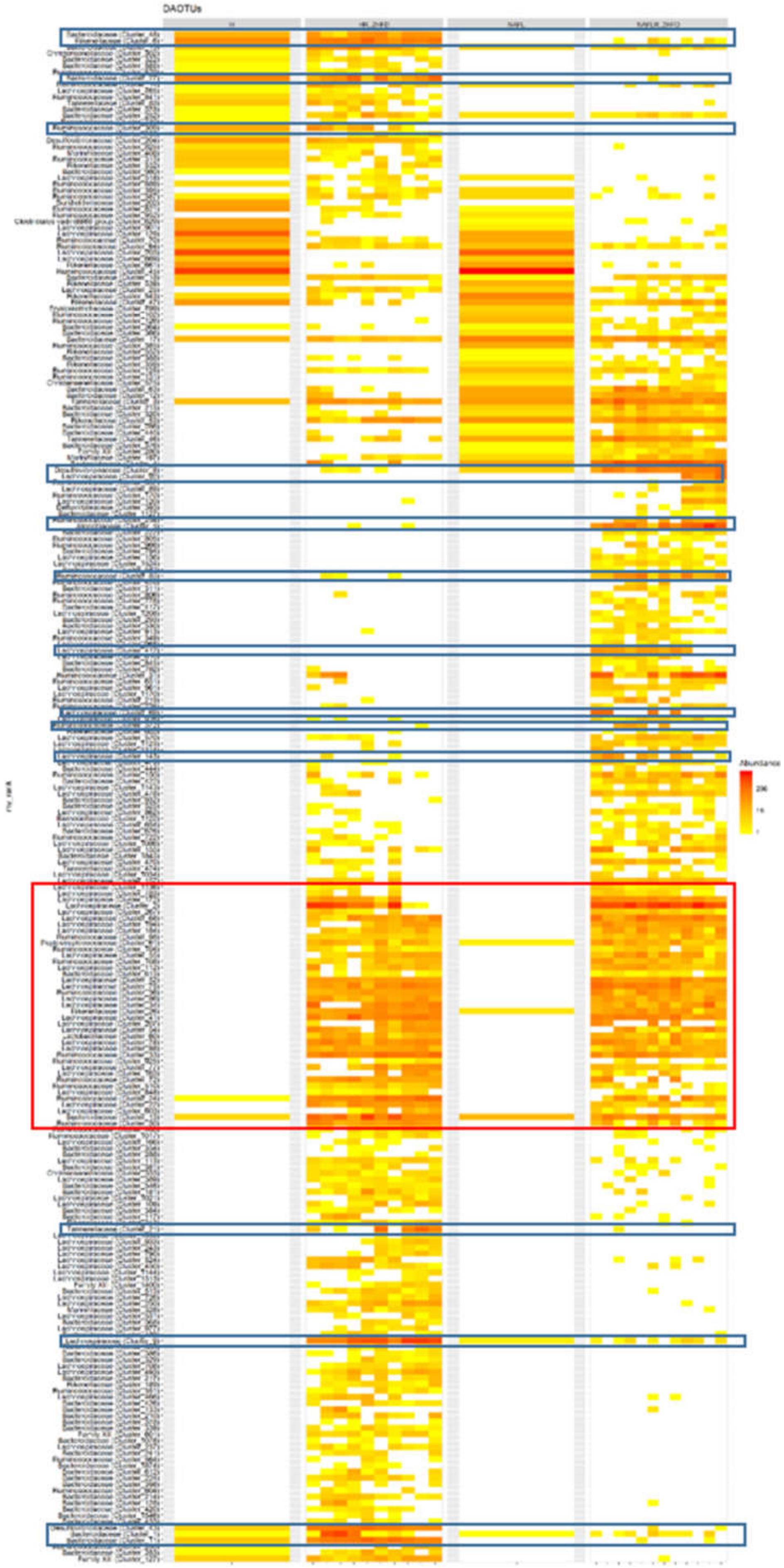
DAOTUs
Abundance

Figure S7. Abundance heatmap representation of differentially abundant OTUs (DAOTUs) between healthy H *inocula*, NAFL *inocula*, HR_2HFD and NAFLR_2HFD groups on day 90, having a large fold change and significant effect size in addition to high relative abundance. Each OTU is a row and is represented according to its taxonomic classification at the family level. OTUs on Figure 11, framed in blue. OTUs corresponding to the effect of 2HFD diet framed in red. HR, healthy human microbiota receiver mice on 2HFD; NAFLR, NAFL patient microbiota receiver mice on 2HFD; 2HFD, high-fructose, high-fat diet.
